# Functional Characterisation and Drug Target Validation of a Mitotic Kinesin-13 in *Trypanosoma brucei*

**DOI:** 10.1371/journal.ppat.1001050

**Published:** 2010-08-19

**Authors:** Kuan Yoow Chan, Keith R. Matthews, Klaus Ersfeld

**Affiliations:** 1 Department of Biological Sciences, University of Hull, Hull, United Kingdom; 2 Centre for Immunity, Infection and Evolution, Institute for Immunology and Infection Research, School of Biological Sciences, University of Edinburgh, Edinburgh, United Kingdom; 3 Hull York Medical School, University of Hull, Hull, United Kingdom; Seattle Biomedical Research Institute, United States of America

## Abstract

Mitotic kinesins are essential for faithful chromosome segregation and cell proliferation. Therefore, in humans, kinesin motor proteins have been identified as anti-cancer drug targets and small molecule inhibitors are now tested in clinical studies. Phylogenetic analyses have assigned five of the approximately fifty kinesin motor proteins coded by *Trypanosoma brucei* genome to the Kinesin-13 family. Kinesins of this family have unusual biochemical properties because they do not transport cargo along microtubules but are able to depolymerise microtubules at their ends, therefore contributing to the regulation of microtubule length. In other eukaryotic genomes sequenced to date, only between one and three Kinesin-13s are present. We have used immunolocalisation, RNAi-mediated protein depletion, biochemical *in vitro* assays and a mouse model of infection to study the single mitotic Kinesin-13 in *T. brucei*. Subcellular localisation of all five *T. brucei* Kinesin-13s revealed distinct distributions, indicating that the expansion of this kinesin family in kinetoplastids is accompanied by functional diversification. Only a single kinesin (TbKif13-1) has a nuclear localisation. Using active, recombinant TbKif13-1 in *in vitro* assays we experimentally confirm the depolymerising properties of this kinesin. We analyse the biological function of TbKif13-1 by RNAi-mediated protein depletion and show its central role in regulating spindle assembly during mitosis. Absence of the protein leads to abnormally long and bent mitotic spindles, causing chromosome mis-segregation and cell death. RNAi-depletion in a mouse model of infection completely prevents infection with the parasite. Given its essential role in mitosis, proliferation and survival of the parasite and the availability of a simple *in vitro* activity assay, TbKif13-1 has been identified as an excellent potential drug target.

## Author Summary

Kinesins represent a class of mechanochemical enzymes that are able to move along microtubule filaments and transport cargo in a directional manner within the cell. Of particular importance are mitotic kinesins, as they ensure the accurate segregation of chromosomes and therefore cell survival. Such kinesins are involved in building and maintaining the mitotic microtubule-based spindle and in chromosome translocation during mitosis. Mitotic kinesins are potentially excellent drug targets because of their roles in an essential process of cell multiplication. Unregulated cell proliferation is associated with diseases such as cancer, but also many infectious diseases. Therefore, the identification of kinesins essential for the proliferation of parasites in the human host offers an attractive prospect for intervention. In our study we present a comprehensive biochemical and cell biological analysis of a mitotic kinesin in the protozoan parasite *Trypanosoma brucei*, causative agent of sleeping sickness in Africa. We show that this kinesin is essential for parasite survival not only in cultured cells but also in mice infected with this parasite and therefore establish this kinesin as a potential drug target in parasitic infections.

## Introduction

Although molecular evolutionary analysis places the branching point of kinetoplastids near the root of the eukaryotic tree, many aspects of their cellular architecture and complexity are nevertheless not hugely divergent from metazoan organisms [1]. One of the most conserved elements between kinetoplastids and other eukaryotes are microtubule-based structures [2]. The sequences of α- and β-tubulin are very similar (∼94% similarity at protein level) to their mammalian orthologues and minor tubulin isotypes, such as γ-tubulin, have also been identified [3]. Although defined by only a small subset of conserved proteins, the axonemal structure in the flagellum of kinetoplastids is also virtually identical to that found in cilia and flagella of mammals. *Trypanosoma brucei* has developed into one of the model organisms to study flagellar assembly and a number of flagellar proteins associated with ciliopathies in humans are conserved in trypanosomes [4], [5], [6], [7], [8], [9]. The kinetoplastid genome project has also revealed the presence of a large number of kinesin motor proteins [10]. Recent comprehensive phylogenetic analyses have identified 41 kinesin family proteins in *T. brucei* [11], [12]. This is similar to the number of kinesins found in mammals, e.g. 45 in humans [13]. The large kinesin family in kinetoplastids reflects the complexity of the microtubule cytoskeleton in these parasites. In addition to the flagellum they possess an elaborate subpellicular microtubule corset, an intranuclear mitotic spindle and possibly a small number of cytoplasmic microtubules involved in vesicle transport [2], [14], [15], [16], [17]. Also, the complex karyotype of *T. brucei*, consisting of three different classes of chromosomes (megabase-, intermediate- and minichromosomes) with a total number exceeding one hundred and the associated unusual segregation patterns might require a substantial number of specific mitotic kinesins [reviewed in 18], [19]. Similar large numbers of kinesins are also found in other protozoa possessing elaborate microtubule structures, such as ciliates or diatoms [12]. Based on comparative sequence analysis, the kinesin superfamily of motor proteins can be subdivided in up to 17 families [11], [12], [20], [21].

Due to the lack of functional analysis of kinesins across a wide range of species it is not easily possible to infer precise function of a kinesin based on its assignment to a particular family. Broadly, families tend to either contain kinesins involved in chromosome segregation, spindle dynamics and transport of membranous vesicles or organelles [22], although some families contain kinesins that do not all share similar functions [21]. The exclusive presence of some families in a subset of species able to build cilia and flagella indicates shared biological functions in relation to these structures [12].

The Kinesin-13 family is unusual because, in contrast to most other kinesins, they do not transport cargo along microtubules but instead are able to depolymerise microtubules at both the plus and minus ends. The only other kinesins known to have a depolymerising activity are yeast Kinesin-8 Kip3p and yeast Kinesin-14 Kar3p [23], [24]. However, in contrast to Kinesin-13, Kip3p and Kar3p only depolymerise microtubules at the plus- or minus-end, respectively, and display a processive, ATP-dependent motility along microtubules. In humans, three Kinesin-13 members have been identified (Kif2a, Kif2b and Kif2c). Kif2c is also known as MCAK (mitotic centromere associated kinesin). All three human Kinesin-13 proteins have mitotic functions [25], [26], although Kif2a has been reported to have an additional role in regulating growth cone dynamics in axons [27]. Mitotic kinesins are of particular interest because they are potential drug targets for diseases where cell proliferation is associated with pathogenicity, such as cancer. Several synthetic, small molecule human kinesin inhibitors with antitumour activities have already entered clinical trials and it is hoped that they will lead to the development of novel anticancer drugs [28], [29], [30], [31]. By analogy, a similar strategy is feasible against parasitic diseases where disease progression and pathogenicity is caused by parasite proliferation in the human host, as is the case e.g. for malaria and sleeping sickness. For this reason and to explore their biological functions we have begun to characterise kinesin motor proteins in *T. brucei*. We were specifically interested in mitotic kinesins. Because most members of the Kinesin-13 family in other organisms have mitotic functions we initially focused on their characterisation.

Comparative sequence analysis has identified five Kinesin-13s in kinetoplastids [12]. This surprisingly large number, which exceeds the number of Kinesin-13s in any other organism where a complete genome is available, could either be caused by a mis-assignment using phylogenetic tools or by extended functionality of these depolymerising kinesins in kinetoplastids. A recent report showing that one member of this family in *Leishmania major* is involved in flagellar length regulation and not in mitosis indicates that functional diversification is the most likely reason for the expansion of this kinesin family in kinetoplastids [32]. Here we report the characterisation of the single mitotic kinesin of the Kinesin-13 family in *T. brucei* (termed TbKif13-1). We show that it is important for the regulation of spindle length during mitosis. We also demonstrate that TbKif13-1 is essential for cell viability in procyclic and bloodstream from *T. brucei* in culture. Importantly for its potential as a drug target, RNAi-mediated protein depletion in a mouse model completely protects from infection.

## Results

### Kinesin-13 motor proteins in *Trypanosoma brucei*

**Figure 1:**
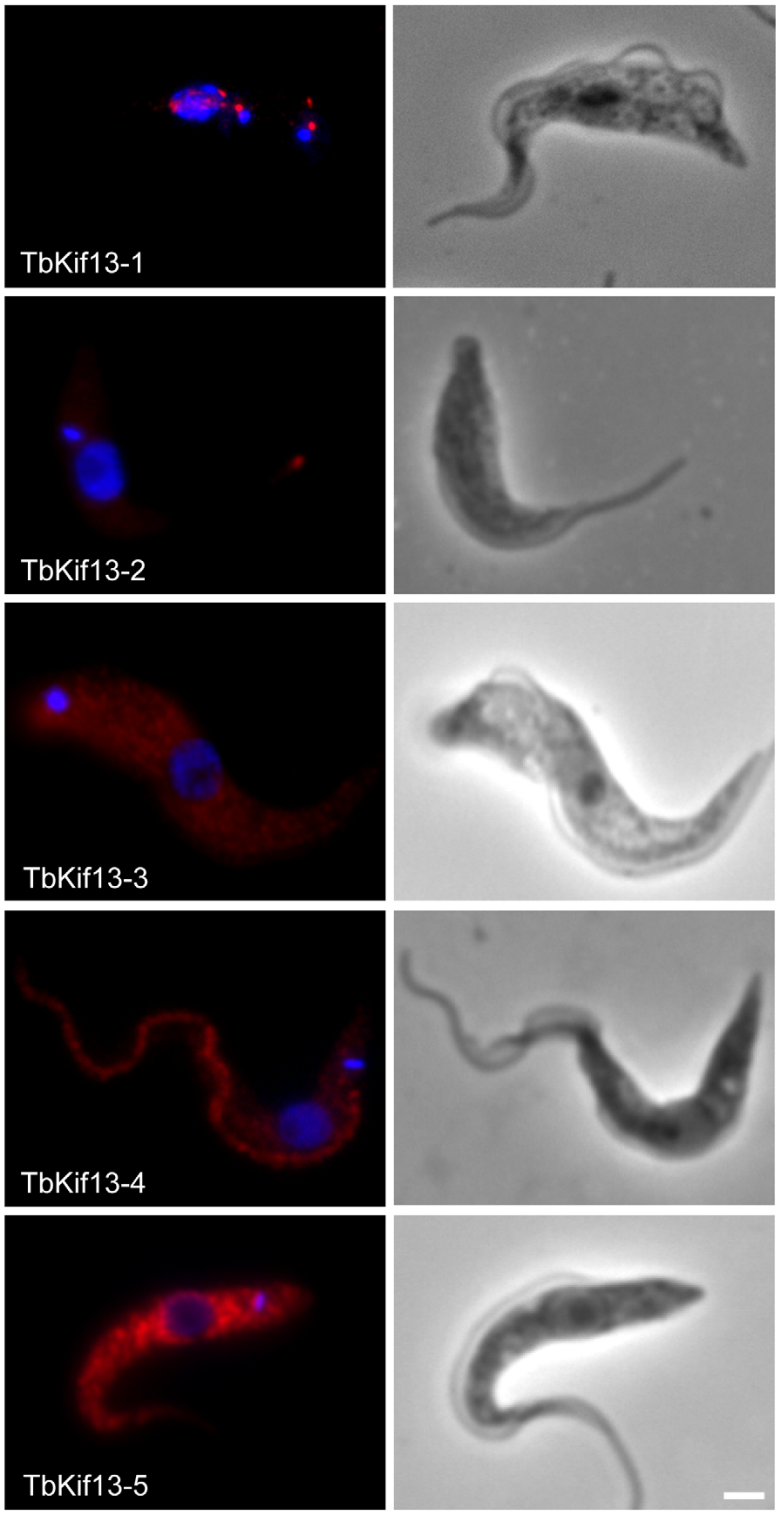
Immunolocalisation of trypanosome Kinesin-13 members. Fluorescence images of procyclic cells stained with anti-Kinesin13 antibodies (red). Nuclear and kinetoplast DNA has been counterstained with DAPI (blue). The corresponding phase contrast images are shown in the right panel. Immunolocalisation of endogenous TbKif13-1, TbKif13-3 and TbKif13-4 was achieved using polyclonal antibodies raised against protein fragments specific to each of the kinesins. The immunolocalisation of TbKif13-2 and TbKif13-5 was done by overexpressing a C-terminal cmyc-tagged recombinant version of the corresponding kinesin employing a tet-inducible expression system. An anti-myc monoclonal antibody was used for the detection of the epitope-tagged proteins. Bar, 2 µm.

A phylogenetic analysis of kinesins in *T. brucei* has assigned five kinesins to the microtubule-depolymerising Kinesin-13 family [12]. This large number is unusual because humans and other eukaryotes have only three or fewer members of this family, most of which are involved in mitotic processes. To investigate whether *T. brucei* Kinesin-13s have more diverse cellular functions we determined the subcellular localisations of all five Kinesin-13s (Fig. 1). We generated polyclonal antibodies against recombinant proteins representing specific regions of each kinesin (Fig. S1). Of the five kinesins only TbKif13-1 localised to the nucleus. Its localisation is similar to the nuclear localisation of the previously identified orthologue LmjKIN13-1 in *Leishmania major* [33]. The other four *T. brucei* Kinesin-13s localised to non-nuclear targets. TbKif13-4 is found along the entire flagellum and TbKif13-3 is homogeneously distributed across the cell body, but is excluded from the flagellum and nucleus. We were unable to detect expression of endogenous TbKif13-2 and TbKif13-5 in procyclic and bloodstream trypanosomes by immunofluorescence or western blotting. To discriminate whether this was due to the failure of the antibodies to detect the proteins or due to the absence, below detection levels, of both proteins, we expressed inducible, cmyc-tagged ectopic copies of both kinesins. After induction, overexpressed TbKif13-2 and TbKif13-5 were detectable by immunofluorescence microscopy and blotting using antibodies against the native proteins and also by anti-myc antibodies (Fig. 1, Fig. S1). Therefore, the most likely explanation of the inability to detect endogenous protein is that both proteins are either not expressed in these two life cycle stages or at extremely low levels. The ectopically expressed TbKif13-2 is localised to the tip of the flagellum and TbKif13-5 is distributed throughout the cell body. A flagellar tip localisation has also been reported for the GFP-tagged, ectopically expressed kinesin LmjKIN13-2 in promastigote *Leishmania major*, a protein very similar in sequence to TbKif13-2 [32]. The distinct localisation of TbKif13-2 at the flagellar tip and the same localisation of its *L. major* homologue suggests that this is the genuine localisation of this kinesin, although in both studies detection was only achieved with an overexpressed, epitope-tagged protein. However, RNAi of this protein in procyclic *T. brucei* caused flagellar lengthening, indicating that it has role at this life cycle stage [32]. Although the localisation and expression of endogenous TbKif13-5 needs to be further examined, both TbKif13-2 and TbKif13-5 are excluded from the nucleus during the entire cell cycle (Fig. S2) and are therefore unlikely to have functions in chromosome segregation. Therefore, the most plausible scenario is that TbKif13-1 is the only nuclear and mitotic Kinesin-13 in *T. brucei*, an organism that undergoes a closed mitosis. None of the kinesins, with exception of TbKif13-1, showed differential expression or localisation during the cell cycle (Fig. S2).

### Cell cycle-dependent expression of TbKif13-1

**Figure 2:**
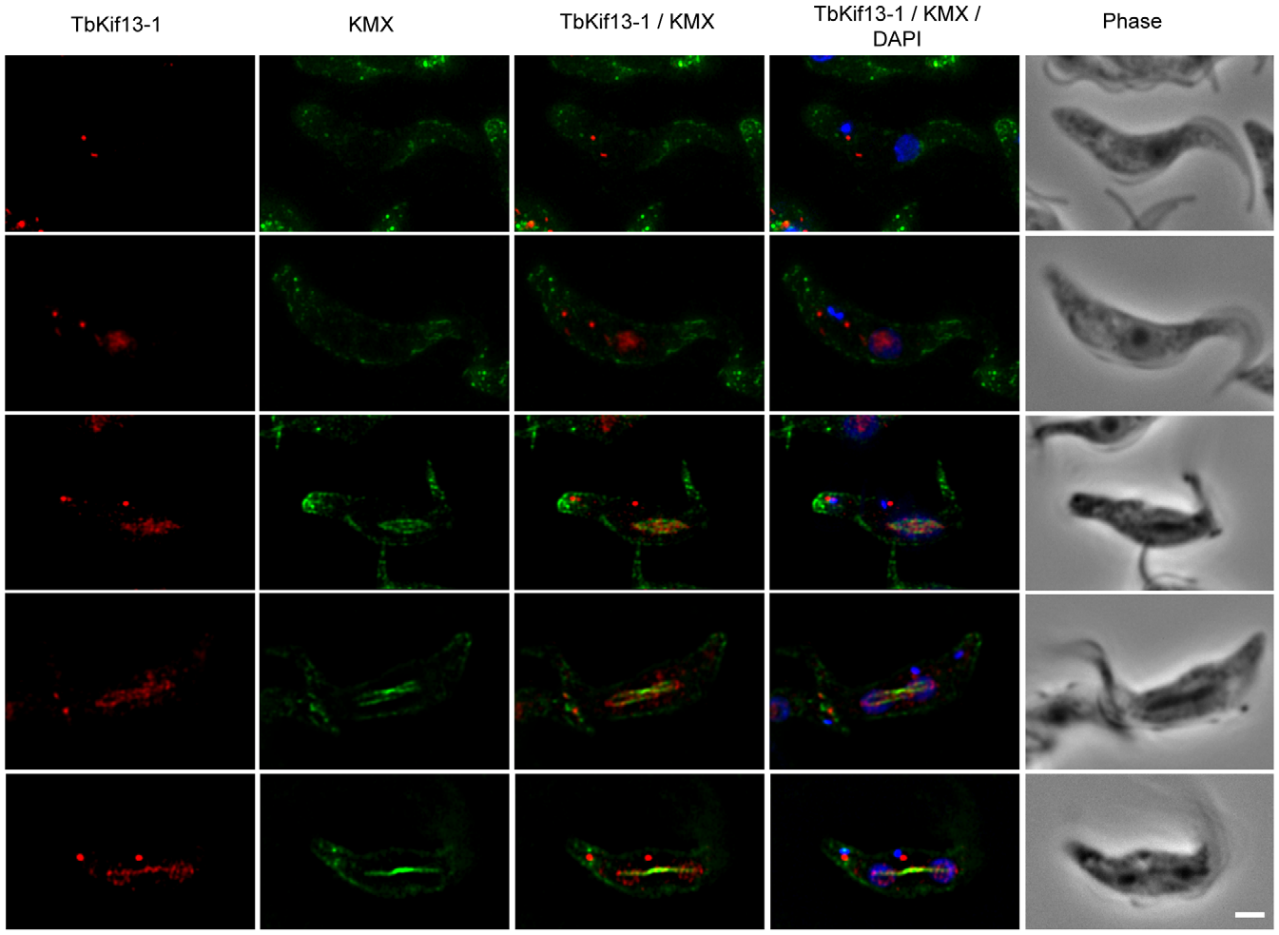
Cell cycle dependent localisation of TbKif13-1. Cellular localisation of TbKif13-1 (red) during the cell cycle. The position of a cell in the cell cycle was assessed by its number of kinetoplasts and nuclei. 1K1N cells are in interphase, 2K1N are either in late G2 or early mitosis and 2K2N cells are in mitosis. The mitotic spindle was stained with the anti-tubulin antibody KMX (green). Nuclear and kinetoplast DNA is stained with DAPI (blue). Bar, 2µm.

**Figure 3:**
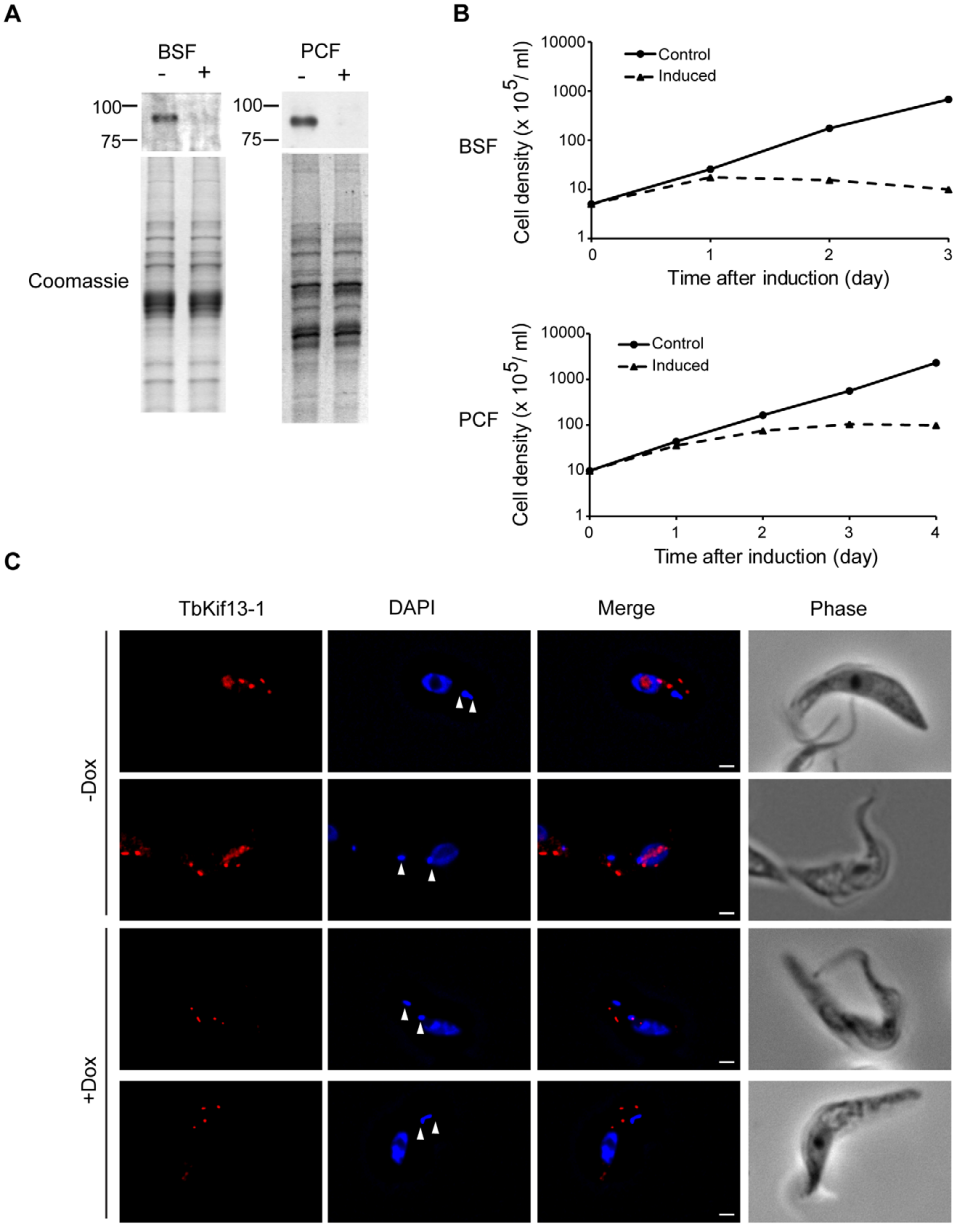
TbKif13-1 is an essential protein in trypanosomes. (A) Western blot on whole cell lysates of bloodstream (BSF) and procyclic (PCF) TbKif13-1 RNAi-depleted cells, either non-induced (−) or induced (+) with doxycycline for 2 days. The Coomassie staining serves as a loading control. (B) A cumulative logarithmic growth curve comparing the growth rates of induced and non-induced (control) procyclic and bloodstream RNAi cells. (C) Fluorescence images of procyclic non-induced (−Dox) or induced (+Dox) for two days. RNAi-mediated depletion results in the loss of nuclear staining, but leaves the cytoplasmic dot-like structures unaffected. The position of the kinetoplasts is indicated by arrowheads. Bar, 2 µm.

Immunofluorescence microscopy on procyclic 427 cells using anti-TbKif13-1 revealed that the nuclear staining of TbKif13-1 is cell cycle-dependent (Fig. 2). TbKif13-1 was not detectable in nuclei of non-dividing interphase cells, recognisable by the 1 kinetoplast/1 nucleus configuration (1K1N). The nuclear staining of TbKif13-1 was detectable in the nuclei from late G2/early M-phase (2K1N) and before the mitotic spindle was visible. Interestingly, the initial signal colocalised with the nucleolus. Upon formation of the mitotic spindle TbKif13-1 colocalised with the spindle structure throughout mitosis. The bulk of the kinesin staining localised to the central, pole-to-pole bundle of spindle microtubules. During late anaphase (Fig. 2, bottom panel) protein could also be detected outside the central spindle, spreading into adjacent chromatin. In addition to the nuclear localisation, the antibodies also revealed two distinct dots inside the cell body, often, but not always, adjacent to the kinetoplast. We observed a duplication of this signal from two dots to four dots in G2-phase or early mitosis (Fig. 3). This additional pattern of TbKif13-1 staining was due to cross reactivity of the antibody with an unknown protein in immunofluorescence, but not Western blotting, and was, in contrast to the nuclear signal, not affected by RNAi-mediated TbKif13-1 depletion. We also introduced an ectopically overexpressed, epitope-tagged copy of TbKif13-1 and did not observe the structures outside the nucleus (Fig. S3).

### TbKif13-1 knockdown results in cell death due to failure of mitosis

To study the function of TbKif13-1 in *T. brucei*, procyclic and bloodstream cells were stably transfected with an RNA interference plasmid construct. RNAi against TbKif13-1 was induced by the addition of doxycycline and resulted in the depletion of TbKif13-1 to undetectable levels by Western blotting and immunofluorescence within 48 hours (Fig. 3). As indicated above, only the nuclear staining of TbKif13-1 was depletable while the staining of the two-dot structures of TbKif13-1 remained unaltered (Fig. 3C). In both life cycle stages, the depletion of TbKif13-1 resulted in growth defects 24 hours post-induction (Fig. 3B). After three days (bloodstream cells) and four days (procyclic cells) cell numbers were reduced by >99% in comparison to the non-induced controls.

**Figure 4:**
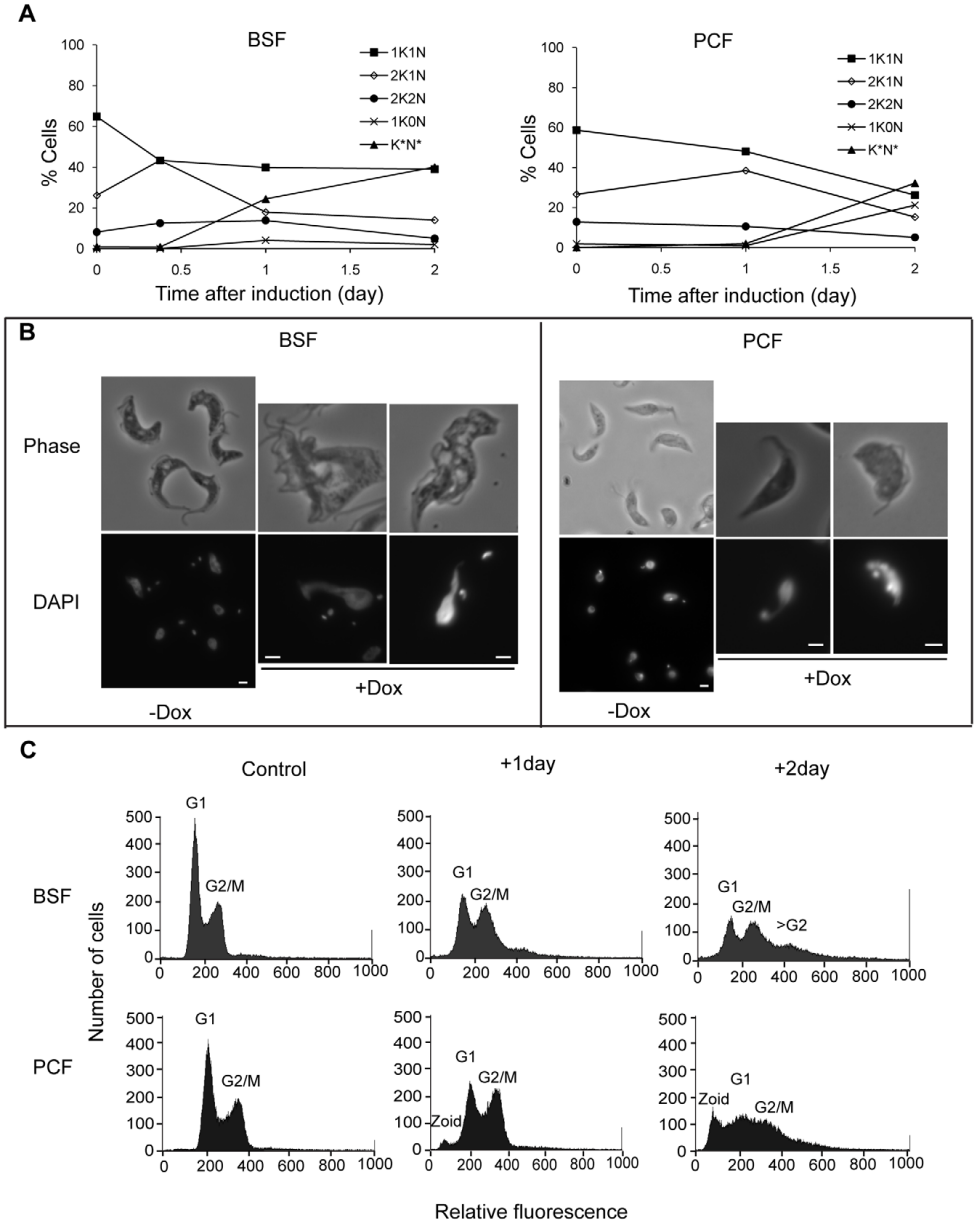
Depletion of TbKif13-1 results in abnormal nucleus and kinetoplast numbers. (A) Analysis of the numbers of kinetoplasts (K) and nuclei (N) in bloodstream (BSF) and procyclic (PCF) cells. (K*N*) denotes cells with morphologically abnormal nuclei and/or >2 kinetoplast. At least 100 cells were counted for each time point. (B) Phase contrast and the corresponding DAPI stained fluorescence images of non-induced (−Dox) and induced (+Dox) cells. Bar, 2 µm. (C) Flow cytometry analysis of non-induced (control) and induced cells treated with doxycycline for one or two days.

The depletion of TbKif13-1 resulted in the accumulation of cells with abnormal morphologies (Fig. 4). In bloodstream cells, depletion resulted in an decrease of 1K1N cells and an increase of 2K1N cells within 9 hours of induction (from 26% to 43%) before declining to 17% of total population in 24 hours after induction (Fig. 4A). After 48 hours of doxycycline addition, 24% of the total cell population consisted of cells with more than 2 kinetoplasts and abnormally shaped nuclei (Fig. 4B). Cells containing more than 2 kinetoplasts indicate failure of cytokinesis. This was further supported by flow cytometry analysis (Fig. 4C). After 48 hours of induction both G1 and G2/M peaks had decreased from 56% (G1) and 38% (G2/M) to 26% (G1) and 33% (G2/M), respectively, with the appearance of a third peak of higher fluorescence (24% of counts, >G2) indicating a DNA content of more than 4n corresponding to cells that had undergone at least two rounds of DNA replication in the absence of cytokinesis.

In procyclic cells, the depletion of TbKif13-1 also resulted in a decrease of 1K1N cells and the accumulation of 2K1N cells (Fig. 4A). At 24 hours post induction, the proportion of 2K1N cells raised from 27% to 38% before declining to 15% after 48 hours. By 48 hours post induction, 25% of the cells were observed to be 1K0N (anucleate zoids) and a further 32% were cells containing abnormal nuclei with either 1 or 2 kinetoplasts. Nuclear abnormalities observed included enlarged and irregular shaped nuclei (Fig. 4B). Flow cytometry analysis showed that after 24 hours of induction, there was a reduction of the G1 peak (55% to 44%) and an increase of the G2/M peak (39% to 44%) (Fig. 4C). This correlates with the observed decrease of 1K1N cells and increase of 2K1N cells. After 48 hours of induction, the G1 and G2/M peaks were further reduced (55% to 35% (G1) and 39% to 28% (G2/M)) and a third peak with a DNA content <2n is apparent, corresponding to the accumulation of zoids in the cell population (21% of counts). Zoid formation was not observed in bloodstream forms, in agreement with other studies showing that defects in mitosis and karyokinesis prevent the completion of cytokinesis only in bloodstream but not in procyclic cells [34], [35], [36].

**Figure 5:**
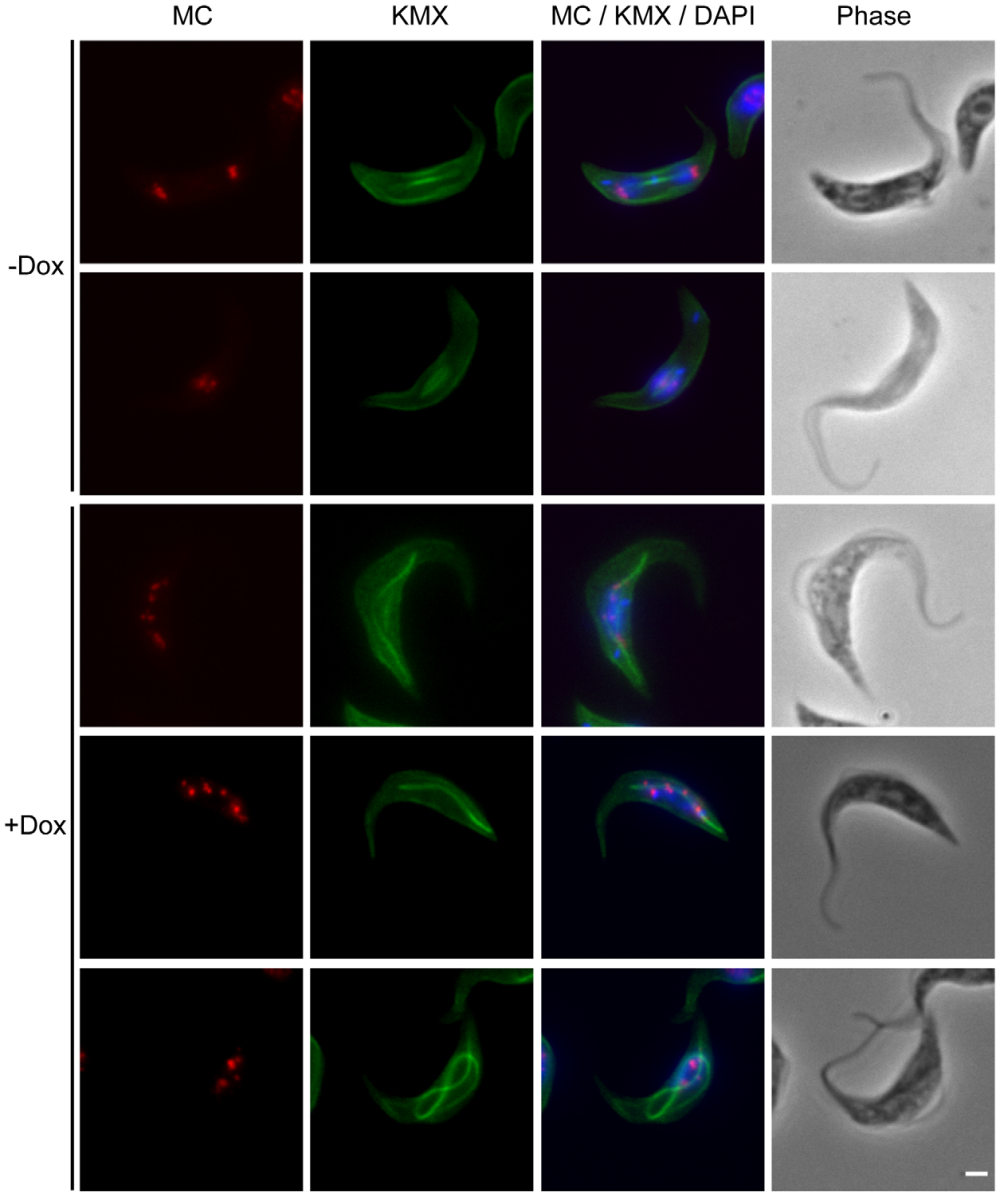
TbKif13-1 depletion results in chromosome segregation defects. Combined immunofluorescence and fluorescent in situ hybridisation (FISH) of procyclic cells using antibody KMX (green) to visualise the mitotic spindle and the 177bp-repeat probe to visualise minichromosomes (red). Shown are representative examples of non-induced and induced TbKif13-1 RNAi cells. Total DNA was stained with DAPI (blue). Phase contrast images of cells are shown in the right panel. Bar, 2 µm.

To assess the effect of TbKif13-1 on genome segregation, FISH analysis was performed using two different DNA probes, one specific to the minichromosomal population (Fig. 5) and another specific to the telomeric regions of all chromosomes (Fig. S4). To demonstrate the correlation of minichromosomal mis-segregation with the appearance of the spindle phenotype, we simultaneously labelled the cells with KMX, an anti-tubulin antibody that preferentially stains the mitotic spindle [37]. During normal mitosis, both the minichromosomal and telomeric signals show the expected symmetrical segregation patterns towards the spindle poles. The depletion of TbKif13-1 and the concurrent appearance of elongated spindle resulted in abnormal segregation patterns of the minichromosomes. Rather than segregating in near-symmetrical patterns to opposite nuclear poles, signals were dispersed and randomly distributed along the length of the mitotic spindle. The severe impact the spindle phenotype has on minichromosomal segregation is also compatible with the unusual mode of microtubule-dependent minichromosomal segregation that was proposed previously [19]. A similar pattern of random segregation as a result of TbKif13-1 depletion was observed for telomeres (Fig. S4).

### Depletion of TbKif13-1 causes abnormal mitotic spindle assembly

**Figure 6:**
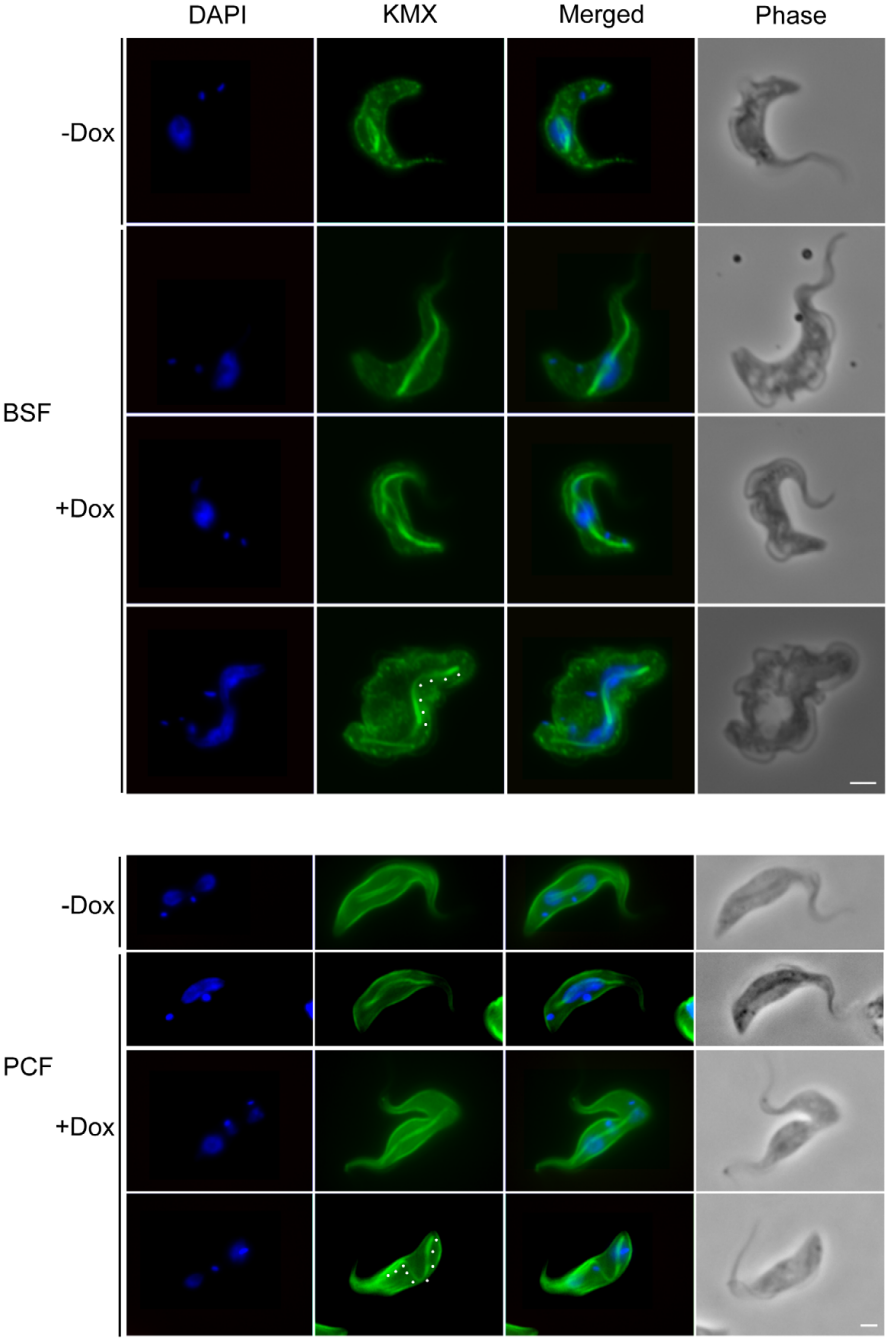
TbKif13-1 depletion causes an extended spindle phenotype. Bloodstream (BSF) and procyclic (PCF) *T. brucei* cells depleted of TbKif13-1 using RNAi were stained with KMX (green) to visualise the mitotic spindle and DAPI to stain nuclear and kinetoplast DNA (blue). Non-induced cells are shown for both life cycle stages to illustrate normal spindle appearance during early (BSF, upper panel, rhomboid spindle) and late mitosis (PCF, upper panel, bifurcated spindle). Note the appearance of bent spindles in late mitosis in BSF and PCF cells (traced with white dots). Bar, 2 µm.

**Figure 7:**
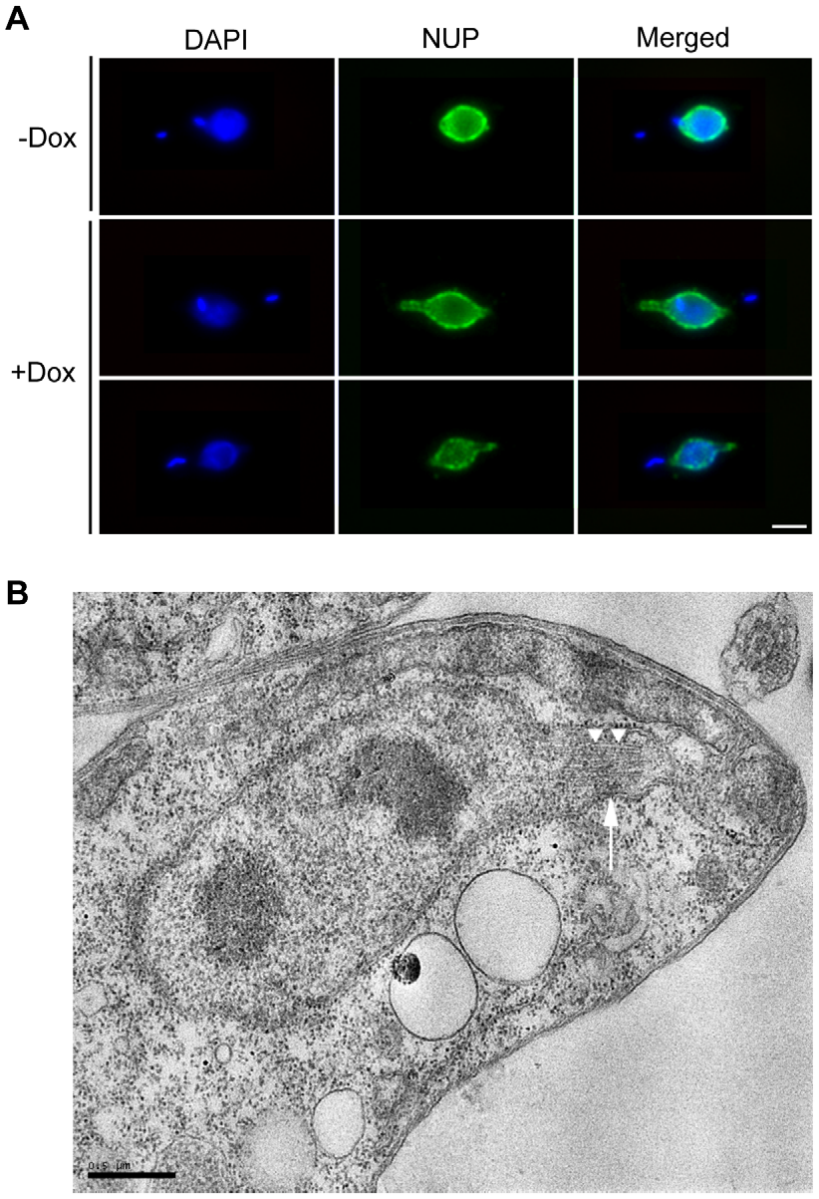
TbKif13-1 depletion results in protrusions of the nuclear envelope. (A) Procyclic TbKif13-1 depleted cells were stained with NUP, an antibody specific to the nuclear envelope. Bar, 2 µm. (B) An EM image of a nuclear cross section of a TbKif13-1 depleted cell. The cell is in mitosis as indicated by the presence of two electron dense regions within the nucleus representing the dividing nucleolus, thus defining the geometry of a dividing nucleus. A protrusion of the nuclear envelope is marked by a white arrow. Several microtubules extend into these protrusions (white arrowheads). Bar 0.5 µm.

Given its colocalisation with the mitotic spindle, the predicted functionality of Kinesin-13s and the appearance of abnormal spindles in FISH experiments, we examined the effect of TbKif13-1 depletion on spindle morphology in detail. In procyclic and bloodstream cells we observed the formation of abnormally long mitotic spindles that occasionally span the entire length of the cell body of the trypanosome (Fig. 5, 6). Other observed spindle phenotypes include bent spindles and abnormally thick spindles. This phenotype is congruent with the predicted function of TbKif13-1 as a microtubule-depolymeriser, leading to longer microtubules in the absence of the protein. It differs from the phenotype described for two mitotic *T. brucei* kinesins TbKin-A and TbKin-B that form part of a chromosomal passenger complex and also co-localise with the mitotic spindle [38], [39]. There, RNAi-mediated depletion prevents the establishment of a mitotic spindle. To test whether the unusually long spindles were still confined within an intact nucleus or actually punctured the nuclear envelope, TbKif13-1 depleted cells were probed with NUP, a monoclonal antibody that recognises a component of the inner nuclear envelope (Fig. 7A, Fig. S5). Although the spindle caused deformations and protrusions of the nuclear envelope, we never observed a discontinuous NUP staining, indicating that the nuclear envelope remained structurally intact. Using transmission electron microscopy, we investigated the ultrastructural appearance of the mitotic phenotype. The protrusions are filled with bundles of microtubules of the mitotic spindle. Again, we noted that the nuclear membrane was intact at the tip of these protrusions (Fig. 7B, Fig. S6). Congruent with the role of TbKif13-1 in spindle length regulation was also the observation that ectopic overexpression of this kinesin led to a block of the cell population in early mitosis and failure to form a recognisable spindle (data not shown).

### TbKif13-1 is essential for trypanosome survival in mice

To test whether TbKif13-1 is essential for parasite infection in a disease model, ten mice were inoculated with the bloodstream TbKif13-1 RNAi cell line. Five of the mice were fed with water containing doxycycline to induce the depletion of TbKif13-1 whilst the remaining five mice were kept as non-induced controls. Blood samples were taken from all ten mice at daily intervals to chart parasitemia (Table 1). Within three days of inoculation, all five mice from the control (i.e. non-depleted) population developed high levels in parasitemia and had to be culled after five days to terminate the experiment. This was in contrast to the doxycycline-fed mice where all five mice remained parasite-free throughout the duration of the experiment. Parasites were not monitored beyond day 5, at which point the control (uninduced) mice had all either died or had reached a humane end point and the experiment had to be discontinued. We cannot exclude that very small numbers of parasites had survived which could subsequently outgrow but these would be likely to be RNAi escape mutants and therefore not informative.

**Table 1:** *In vivo* analysis of TbKif13-1 depletion by RNAi.

Doxycyclin	Mice	Day 1	Day 2	Day 3*	Day 4*	Day 5
Control	M1	ND	positive	9.40E+07	3.75E+08	culled
	M2	ND	positive	4.70E+07	2.50E+08	culled
	M3	ND	positive	4.70E+07	2.50E+08	culled
	M4	ND	positive	2.35E+07	1.87E+08	culled
	M5	positive	positive	9.40E+07	2.50E+08	culled
Induced	M1	ND	ND	ND	ND	ND
	M2	ND	ND	ND	ND	ND
	M3	ND	ND	ND	ND	ND
	M4	ND	ND	ND	ND	ND
	M5	ND	ND	ND	ND	ND

*Parasitemia (parasites/ml of blood).

ND = no parasites detected, positive = parasites detected.

### Biochemical analysis of TbKif13-1

Our cell biological analysis strongly supported the phylogenetic assignment of TbKif13-1 to the Kinesin-13 family. A careful manual alignment also showed the conservation of motifs critical for the depolymerising activity (Fig. S7) [40], [41], [42].

To complement the *in situ* analysis of TbKif13-1 we proceeded to characterise the enzymatical properties of this protein. This analysis was also essential to determine the suitability of this kinesin as a potential drug target because large-scale inhibitor screens are based on *in vitro* inhibition of the kinesin ATPase activity. It should be noted that the assays described below have been done with tubulin preparations of bovine origin, demonstrating that trypanosome tubulin, which is virtually impossible to purify in large quantities, is dispensable to conduct such tests [also see 43].

**Figure 8:**
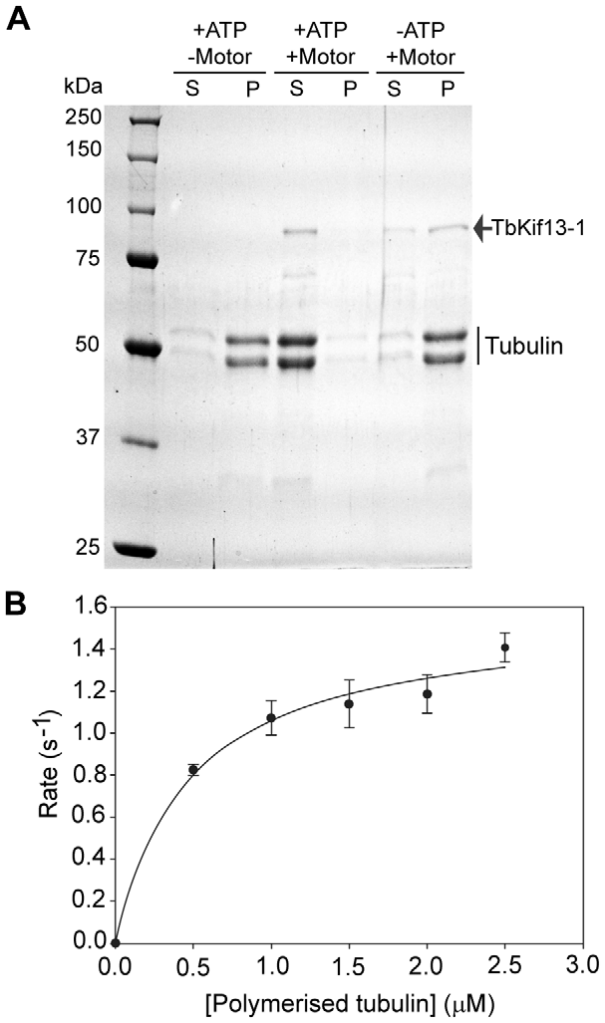
*In vitro* biochemical analysis of recombinant TbKif13-1. (A) Microtubule depolymerisation assay of TbKif13-1 in the presence (+) or absence (−) of ATP/kinesin. S, supernatant fraction; P, pellet fraction. (B) ATPase activity of TbKif13-1 is stimulated by microtubules. Each data point represents three independent measurements. A curve of best fit was drawn using the Michaelis-Menten equation in SigmaPlot V11.0.

To assay the depolymerising properties of TbKif13-1, purified recombinant full-length kinesin was tested in a microtubule sedimentation assay (Fig. 8A). Taxol-stabilised microtubules were incubated with kinesin in the presence or absence of ATP. The depolymerisation of the microtubules by TbKif13-1 was qualitatively examined by the shift of tubulin from the pellet fraction (representing polymerised microtubules) to the supernatant fraction (representing soluble, depolymerised tubulin) using SDS gel electrophoresis. In the presence of ATP and TbKif13-1, more than 95% of total tubulin was found in the supernatant fraction. In the absence of ATP more that 95% of total tubulin was found in the pellet fraction. These data confirm that TbKif13-1 is an ATP-dependent microtubule depolymerising kinesin.

To determine the predicted microtubule-stimulated ATPase activity of TbKif13-1, the steady state ATPase rate was measured at various microtubule concentrations (Fig. 8B). The calculated maximum ATPase activity rate (k_cat_) of TbKf13-1 was 1.50±0.05 s^−1^ (average ± std. dev.). This is a similar rate to that observed for the single Kinesin-13 in *Plasmodium falciparum* (k_cat_ 1.8 s^−1^), but also in a similar range to depolymerising kinesins in other organisms [44], [45], [46]. Initial reactions rates were strongly stimulated by the presence of taxol-stabilised microtubules. The basal ATPase activity in the absence of microtubules was not significantly above background levels with no kinesin added (Fig. S8).

## Discussion

This report represents the first survey of the entire Kinesin-13 family in the kinetoplastid group and identifies the subcellular localisation of all family members coded by the *T. brucei* genome [12]. Of the five Kinesin-13s, only one kinesin has a nuclear localisation (TbKif13-1) while the others were associated with the flagellum (TbKif13-2 and TbKif13-4) and the cell body (TbKif13-3 andTbKif13-5).

The presence of five Kinesin-13s is unique to kinetoplastids. Humans and *Drosophila* possess three Kinesin-13s each and most protozoan genomes known to date contain only a single member of this family [11]. Even in the ciliate *Tetrahymena thermophila*, which, with 78 kinesin sequences, has more kinesins than any other sequenced organism, only three Kinesin-13s were classified [12], [47]. In *S. cerevisiae* and *S. pombe*, Kinesin-13s are completely absent, although they contain members of families Kinesin-8 and Kinesin-14 which are also able to depolymerise microtubules, albeit in a different manner to Kinesin-13s [23], [24], [48]. Kinesin-13s in metazoans have predominantly mitotic functions. In humans, all three Kinesin-13s are involved in mitosis, although one of them (Kif2A) has additional cellular functions [26], [27]. The diverse subcellular localisations of the *T. brucei* Kinesin-13s indicate that this restricted functionality does not apply in this organism. Rather, the expansion of the number of Kinesin-13s is accompanied by functional divergence. This is in contrast to the situation in the protozoan parasite *Giardia lamblia* where the single Kinesin-13 has functions both in flagellar dynamics and mitosis [49]. The only Kinesin-13 identified in the algae *Chlamydomonas rheinhardii* functions in flagellar assembly and disassembly and has no apparent function in mitosis [50]. In *Arabidopsis thaliana*, at least one of the two Kinesin-13s is also non-mitotic and involved in Golgi-associated functions [51], [52]. The distinct non-nuclear localisations of TbKif13-2, TbKif13-3, TbKif13-4 and TbKif13-5 proteins in *T. brucei* provide an excellent opportunity to study the functional diversity of the Kinesin-13 family.

Immunolocalisation showed that TbKif13-1 has a nuclear localisation which was restricted to the mitotic phase of the cell cycle. A similar distribution has been reported for its functional homologue in *Leishmania*, LmjKIN13-1, and in this kinetoplastid the ubiquitin/proteasome pathway is implicated in the cell cycle-dependent regulation of expression [33].

The function of TbKif13-1 was analysed using RNAi-mediated protein depletion in bloodstream and procyclic cell lines. *T. brucei* undergoes a closed mitosis and the spindle develops inside the nucleus and karyokinesis precedes cytokinesis [reviewed in 53]. In both life cycle stages, the depletion of TbKif13-1 resulted in the formation of extremely long, distorted and bent spindles, leading to chromosome segregation defects. Given that Kinesin-13s are able to depolymerise microtubules at both the plus- and minus- end, a spindle elongation phenotype is expected. Although aberrant spindle dynamics are observed in other organisms as well [54], *in vivo* RNAi-depletion of MCAKs in metazoan organisms is associated with less severe phenotypes, in contrast to MCAK depletion done in spindle reconstitution extracts [55]. This has been attributed to observations showing that an increase in the concentration of free tubulin dimers leads to the downregulation of further translation, thereby shifting microtubule dynamics towards depolymerisation and counteracting the lack of enzymatic, kinesin-mediated depolymerising activity [56], [57], [58]. Consequently, the severe spindle elongation phenotype indicates that this mechanism of tubulin translational auto-regulation is most likely not operational in trypanosomes. An additional factor for the extreme phenotype observed in *T. brucei* could be that TbKif13-1 is possibly the only mitotic depolymerising kinesin, whereas in humans and *Drosophila* several Kinesin-13s and depolymerisers of the Kinesin-8 families have potentially overlapping functions and are, to some extent, able to compensate for the loss of a single kinesin [59], [60]. *T. brucei* does not have members of the Kinesin-8 family. Recently, a member of the Kinesin-14 family (Kar3Cik1) in *S. cerevisiae* has also been identified as a microtubule-depolymeriser [61]. Other members of this family, however, are involved in microtubule-crosslinking and -sliding and do not show depolymerising activity [62]. *T. brucei* has two kinesins of the Kinesin-14 family [12]. One of these kinesins has been reported to be involved in acidocalcisome maintenance and the second member has not been characterised yet [43].

Biochemical analyses of purified full length TbKif13-1 shows that this protein depolymerises microtubules in an ATP dependent manner similar to Kinesin-13s in other organisms [63], [64]. We show that enzymatically active, recombinant kinesin can be purified from *E. coli* and that bovine tubulin can be used both for depolymerising and ATPase assays. In summary, we demonstrate the essentiality of TbKif13-1 for *T. brucei* and analyse the biological and biochemical basis of its function. It establishes this kinesin as a potential drug target against sleeping sickness.

## Materials and Methods

### Ethics statement

All animal experiments were carried out in accordance with the ethical rules for animal husbandry of the University of Edinburgh and a UK Home Office license (held by K.R.M) granted for this research as covered by the Animals (Scientific procedures) Act 1986.

### Cell culture

The *T. brucei* bloodstream Lister 427-derived “single marker” strain, expressing Tet-repressor and T7 RNA polymerase, was grown in HMI-9 medium at 37°C, 5% CO_2_ in the presence of G418 at 0.5 µg ml^−1^ [65], [66]. Procyclic strains 427 and 29-13 were maintained in SDM-79, at 28°C [66], [67]. The 29-13 cell line, expressing T7 RNA polymerase and Tet repressor, was grown in the presence of 50 µg ml^−1^ of hygromycin and 15 µg ml^−1^ of G418 [66]. Cell growth was monitored using a CASY cell counter (Roche Innovatis AG, Germany).

### Generation of TbKif13-1 RNAi cell lines

The RNAi construct, pFC4-TbKif13-1 was made using the stem-loop pFC4 vector which allows for the tetracycline inducible production of dsRNA with the use of a single T7 promoter [68]. A 597 bp DNA fragment of the TbKif13-1 open reading frame was PCR-amplified using the sense primer 5′-GCGGATCCAAGCTTAAGCAGATGTTCGTGTCTTTCTAC-3′and anti-sense primer 5′-GCCTCGAGTCTAGAACGGCTTTTCTTTAACTCCTTCACA-3′. The PCR fragment was cloned into the RNAi vector using the restriction sites HindIII, XbaI, XhoI and BamHI (underlined). The resulting construct was linearised with NotI and transfected into procyclic or bloodstream cell by electroporation. Transformants were selected with blasticidin (10 µg ml^−1^ for procyclic and 3 µg ml^−1^ for bloodstream cells). RNAi was induced by the addition of 1 µg ml^−1^ of doxycycline. Suitable RNAi fragments were selected using RNAit software to avoid off-target effects [69].

### Production of polyclonal antibody against *T. brucei* Kinesin-13 proteins

The nomenclature of *T. brucei* Kinesin-13s was adopted from the nomenclature introduced for members of this family in *Leishmania* [32], [33].

Fragments of the open reading frames of TbKif13-1 (residues 486–687, acc no: Tb09.160.2260), TbKif13-2 (amino acid residues 364–683, TriTrypDB acc no: Tb11.02.2260), TbKif13-3 (residues 342–561, acc no: Tb11.02.2970), TbKif13-4 (residues 581–780, acc no: Tb927.4.3910) and TbKif13-5 (residues 545–712, acc no: Tb11.02.0790) were PCR-amplified using a proofreading polymerase (Accusure, Fermentas) and primers specified in Table S1. All fragments were checked against the *T. brucei* genome sequence database (http://www.genedb.org) using Blast to ensure their specificity. The PCR fragments were cloned into the pTrcHisC vector (Invitrogen) using restriction sites BamHI and EcoRI, resulting in the generation of expression constructs with an N-terminally 6×histidine tagged kinesin fragments. Recombinant proteins were expressed in *E. coli* BL21, cells lysed under native (TbKif13-4, TbKif13-3, Tbif13-1) or denaturing (TbKif13-2, TbKif13-5) buffer conditions using a French Press and affinity-purified on cobalt metal resin (Talon, BD Biosciences). The purified recombinant proteins were used to raise rabbit polyclonal antibodies (Yorkshire Biosciences). Polyclonal antibodies were affinity-purified using the recombinant kinesin fragments, coupled to CNBr-activated Sepharose 4B (Amersham Biosciences). Bound protein was eluted with glycine buffer (100 mM glycine-HCl, 100 mM NaCl, pH 2,5). The purified antibodies were diluted 1∶25 for immunofluorescence and 1∶1000 for chemiluminescence-based Western blots.

### Generation of TbKif13-2^myc^ and TbKif13-5^myc^ cell line

Constructs of the C-terminally 2×myc-tagged TbKif13-2^myc^ and TbKif13-5^myc^ kinesins were generated using the tetracycline inducible expression plasmid pHD1484 [70]. The entire open reading frames of TbKif13-2 and TbKif13-5 were PCR-amplified using primers specified in Table S2. The PCR fragments were cloned into the pHD1484 using the restriction sites Apa1 and BamH1. The NotI-linearised expression construct was electroporated into the procyclic strain 449 cell line, expressing Tet-repressor. The selection of stable transfectants, integrated into the ribosomal RNA gene locus was done with hygromycin at 50 µg ml^−1^. Expression of tagged kinesins was induced by the addition of 1 µg ml^−1^ of doxycycline.

### Immunofluorescence microscopy

*T. brucei* cells were fixed in suspension with 3.6% formaldehyde and processed as described [71]. In addition to the rabbit anti-kinesin antibodies, other primary antibodies used in this study were mouse monoclonal anti-β-tubulin antibody KMX [37] to visualize the mitotic spindle, mouse monoclonal anti-nuclear envelope antibody NUP [17] and mouse mAb anti-cmyc (clone 9E10, ECACC). Cells were examined on an Olympus IX71 epifluorescence microscope equipped with a CCD-camera (F-View, Olympus). Images were pseudo-coloured and assembled in Adobe Photoshop CS4.

### Electron microscopy

Procyclic trypanosomes were processed for electron microscopy as described, except that thin sections were not post-stained [72]. Samples embedded in epoxy resin were thin-sectioned and examined on a Jeol 2010 electron microscope operating at 120 KeV. Images were recorded using a Gatan UltraScan 4000 CCD camera.

### Fluorescent *in situ* DNA hybridisation

Visualisation of telomeres by FISH and combined immunofluorescence with KMX and minichromosomal FISH was done essentially as described, except that the telomeric oligonucleotide (TTAGGG)_5_ was synthetically labelled with digoxigenin (MWG) [17], [73].

### Fluorescence flow cytometry analysis

Cells were processed for flow cytometry analysis exactly as described [71] and analysed on a FACS Calibur flow cytometer using CellQuest software (Becton Dickson).

### Expression and purification of active, full length TbKif13-1 in *E. coli*

The entire ORF of TbKif13-1 was PCR amplified using primers 5′-GCGGATCCTCGCGAG TGGGAATTAAAGCTGGT-3′ and 5′-CGAAGCTTCTAAATCCCGTTTTGCTCGAGAC-3′and ligated into the pTrcHisC vector (Invitrogen) using restriction sites BamHI and EcoRI (underlined), resulting in the generation of an expression construct coding for a N-terminally 6×histidine-tagged TbKif13-1 protein. The recombinant full length TbKif13-1 was expressed in *E. coli* BL21 (Promega) and purified using metal affinity resins by BD biosciences (BD Talon). Purification conditions were specifically adjusted to optimize expression of enzymatically active kinesin [74]. BL21 cells expressing TbKif13-1 at low levels were grown at 37°C in a shaking incubator without additional induction by IPTG until cell density at 600 nm was 1.0. Bacteria were cooled to 4°C and harvested by centrifugation at 2,500 g for 10 minutes. Bacterial pellets were then resupended in lysis buffer (100 mM PIPES, 100 mM NaCl, 1 mM MgCl_2_, 10 mM imidazol, 1 mM ATP, 1mM β-mercaptoethanol, 0.2 mM PMSF, 1∶200 dilution of Protease Inhibitor Cocktail (Sigma-P8340), pH 6.9) and lysed via two passages at 1000 kPsi through a French press. The lysate was centrifuged at 14,000 g for 10 minutes at 4°C and the supernatant was incubated with BD Talon resin at 4°C for 20 minutes before being washed twice with 10 bed volumes of wash buffer (100 mM PIPES, 100 mM NaCl, 1 mM MgCl_2_, 10 mM imidazol, 0.01 mM ATP, 1mM β-mercaptoethanol, 0.2 mM PMSF, pH 6.9). Protein was eluted in elution buffer (100 mM PIPES, 100 mM NaCl, 1 mM MgCl_2_, 250 mM imidazol, 0.01 mM ATP, 1mM β-mercaptoethanol, pH 6.9). Glycerol was added to the peak fractions to a final concentration of 20% (v/v). Aliquots of 50µl were snap-frozen in liquid nitrogen and stored at −80°C. The concentration of the purified TbKif13-1 was determined using the BCA protein determination kit (Sigma).

### Microtubule depolymerisation assay

The assay was performed as described [75]. Purified bovine tubulin (Cytoskeleton Inc.) was assembled at 37°C for 30 minutes in PME buffer (80 mM PIPES, 2 mM MgCl_2_, 0.5 mM EGTA, 1 mM DTT, 1 mM GTP, pH 6.9) containing 10 µM taxol (Sigma). Polymerised microtubules were harvested via centrifugation at 250,000 g for 15 minutes at 30°C. The polymerised microtubules were subsequently resuspended to a final concentration of 2.5 µM in MT buffer (80 mM PIPES, 50 mM KCl, 2 mM MgCl_2_, 0.5 mM EGTA, 1 mM DTT, 0.5 mM GTP, 5 µM Taxol, 2% glycerol, pH 6.9) and then incubated with 1.4 µM purified TbKif13-1 in the presence or absence of 1.5 mM ATP for 30 minutes at 28°C and centrifuged at 250,000 g for 10 minutes at 28°C. The supernatant and pellet were analysed by SDS-PAGE.

### ATPase assay

The coupled pyruvate kinase/lactate dehydrogenase steady-state ATPase assay was performed as described [76]. Purified bovine tubulin was assembled at 37°C for 30 minutes in PME buffer (80 mM PIPES, 2 mM MgCl_2_, 0.5 mM EGTA, 1 mM DTT, 1 mM GTP, pH 6.9) containing 10 µM taxol. The polymerised tubulin was added to the ATPase assay at concentrations ranging from 0.5 µM to 2.5 µM. The ATPase assay consisted of 50 mM Tris-acetate, 1 mM MgCl_2_, 1 mM DTT, 1 mM ATP, 3 mM PEP, 0.2 mM NADH, 6.5 µM taxol, 6 mM PIPES, 5 mM NaCl, 12.5 mM imidazol, 13.59 units ml^−1^ lactate dehydrogenase, 11.2 units ml^−1^ pyruvate kinase, 0.42 µM purified TbKif13-1, 1% glycerol, pH 7.5. The initial ATPase rates were determined at 25°C using the change in absorbance at 340 nm. Data analysis was done with SigmaPlot V11.0 using the Michaelis-Menten equation 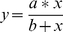, where a is the maximum ATPase rate and b is the K_m_ value of TbKif13-1 (for details see http://www.proweb.org/kinesin/Methods/ATPase_assay.html).

### In vivo analysis of RNAi-mediated TbKif13-1 depletion

Ten male age-matched MF1 mice were inoculated i.p. with 35,000 trypanosomes in a volume of 200 µl HMI-9 and split into two groups. One group of 5 mice was provided with doxycycline (200 µg ml^−1^ in 5% sucrose) in their drinking water immediately post-inoculation, whereas the other group of five mice was supplied with drinking water containing 5% sucrose only. Parasite numbers were scored over 5 days by the rapid matching method of Herbert and Lumsden [77] and animals culled using defined humane end points as specified in the relevant UK Home office license.

## Supporting Information

Figure S1 Validation of anti-kinesin antibodies. (A) Western blot using rabbit pre-immune sera (a), immune sera (b) and affinity purified antibody (c) on whole cell lysates of procyclic *T. brucei*. Note that the purified antibodies against TbKif13-2 and TbKif13-5 do not recognise their cognate antigen. (B) Western blot analysis using antibodies against TbKif13-2 and TbKif13-5 and anti-myc antibodies on wild-type procyclic (PCF), wild-type bloodstream (BSF) and procyclic cell lines transformed with the TbKif13-2^myc^ or TbKif13-5^myc^ expression vector. The lane marked (+) represents cells where expression was induced with doxycycline while the lane marked (−) represents non-induced cells. This demonstrates that the anti-TbKi13-2 and anti-TbKif13-5 antibodies do recognise their respective antigens when overexpressed from an inducible ectopic locus.
(1.52 MB PDF)

Figure S2 Immunolocalisation of trypanosome Kinesin-13s at different cell cycle stages (red signal). Shown are trypanosome cells in interphase (1K1N), in early (2K1N) and late mitosis (2K2N). The immunolocalisation of endogenous TbKif13-3 and TbKif13-4 was achieved using polyclonal antibodies raised against protein fragments specific to each of these two kinesins. The immunolocalisation of TbKif13-2 and TbKif13-5 was done by overexpressing a C-terminal cmyc-tagged recombinant version of the corresponding kinesins employing a tet-inducible expression system. An anti-cmyc monoclonal antibody was used for the detection of the epitope-tagged proteins. Nuclear and kinetoplast DNA has been stained with DAPI (blue). Bar, 2 µm.
(3.56 MB PDF)

Figure S3 Ectopic, cmyc-tagged TbKif13-1 overexpression results in an exclusive nuclear staining. Fluorescent images of procyclic cells overexpressing a full length, ectopic copy of TbKif13-1 (red). DNA was counterstained with DAPI (blue). Bar, 2 µm.
(0.98 MB PDF)

Figure S4 TbKif13-1 depletion results in chromosome segregation defects. Fluorescence *in situ* hybridisation (FISH) of procyclic cells using DNA probes (green) specific to the telomeric repeats of trypanosome chromosomes. The DNA of the kinetoplast and nucleus was stained with DAPI (blue). Shown are representative examples of induced (+Dox) and non-induced (−Dox) TbKif13-1 RNAi cells. Bar, 2 µm.
(0.78 MB PDF)

Figure S5 TbKif13-1 depletion results in abnormally shaped nuclei. Immunofluorescence images of induced (+Dox) and non-induced (−Dox) TbKif13-1 RNAi cells. The staining by NUP, detecting a protein of the inner nuclear envelope, is shown in green while the DNA of the kinetopast and nucleus is shown in blue. Bar, 2 µm.
(3.94 MB PDF)

Figure S6 Transmission electron microscopy of TbKif13-1 RNAi depleted cells. Electron microscopy images of nuclear cross sections of TbKif13-1 depleted cells showing protrusions to its nuclear envelope (panels A–D). Panel B is an enlargement of the nuclear protrusion visible in panel A. Notice the presence of microtubule bundles in the nuclear extensions.
(9.71 MB PDF)

Figure S7 TbKif13-1 is a Kinesin-13 family member. Sequence alignment of the motor domain of TbKif13-1 against known and putative Kinesin-13 proteins. The sequences highlighted in yellow are Kinesin-13 specific motifs [1], [2]. The numbers at the left hand side of the panel denotes the relative position of the residue from the N-terminus of each corresponding protein. The corresponding GenBank accession numbers for each corresponding proteins are NP_701793 (PfKinI, *Plasmodium falciparum*), EAA40236 (GlKinesin-13, *Giardia lamblia*), BAA02165 (MmKif2, *Mus musculus*), AAB17358 (CgMCAK, *Cricetulus griseus*), NP_566534 (AtKlp3, *Arabidopsis thaliana*), NP_047029 (LmjKIN13-1, *Leishmania major*).
(4.56 MB PDF)

Figure S8 Detailed analysis of TbKif13-1 ATPase activity. The graph shows the absorbance changes of the ATPase assay over time. Rate calculations of ATPase activity for TbKif13-1 are exemplified for the presence of 1 µM tubulin. The rate of NADH depletion was obtained by measuring absorbance change at 340 nm, where one mol of oxidised NADH corresponds to the production of one mol of ADP. The ATPase rate was obtained by dividing the change of OD_340_ min^−1^ by extinction coefficient 6.22×10^−3^ µM^−1^ of NADH. The resulting ATPase activity was corrected by the background ATPase activity. The resulting net ATPase activity was divided by the concentration of TbKif13-1 and subsequently divided by 60 s min^−1^ to convert to the ATPase rate (s^−1^) used in Fig. 7B. Also shown in the graph are the rate of absorbance change at 340 nm of the assay in the presence and absence of TbKif13-1 and microtubules, respectively. The error bars in the graph represent the standard deviation of each data point after triplicate measurements.
(0.43 MB PDF)

Table S1 Primer sequences used to generate protein expression constructs for recombinant protein production
(0.16 MB PDF)

Table S2 Primer sequences used in the generation of cmyc-tagged kinesin expression constructs
(0.16 MB PDF)
