# Supplementary figures and images for: Functional Characterisation and Drug Target Validation of a Mitotic Kinesin-13 in Trypanosoma brucei
Source: PLoS Pathog. 2010 Aug 19;6(8):e1001050. doi: 10.1371/journal.ppat.1001050 (PMC2924347; doi:10.1371/journal.ppat.1001050)

**A**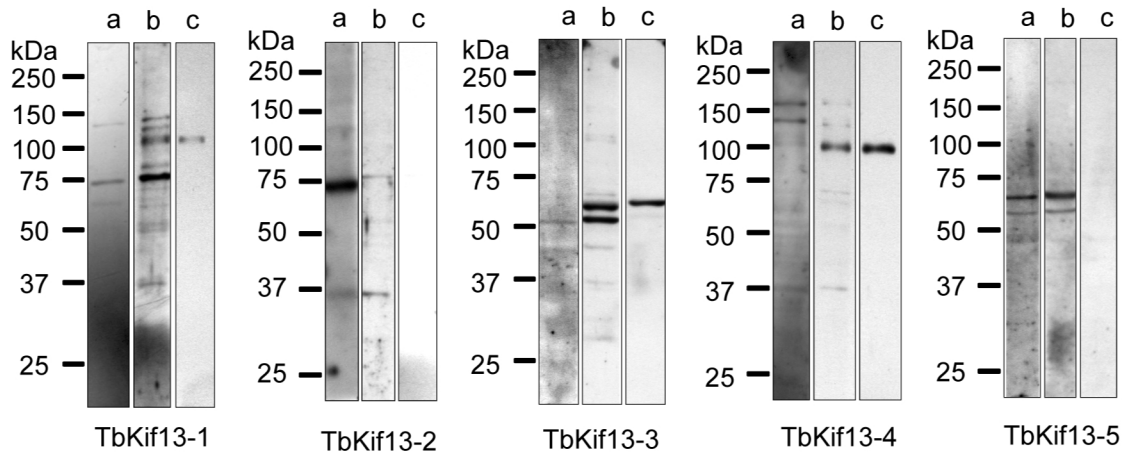**B**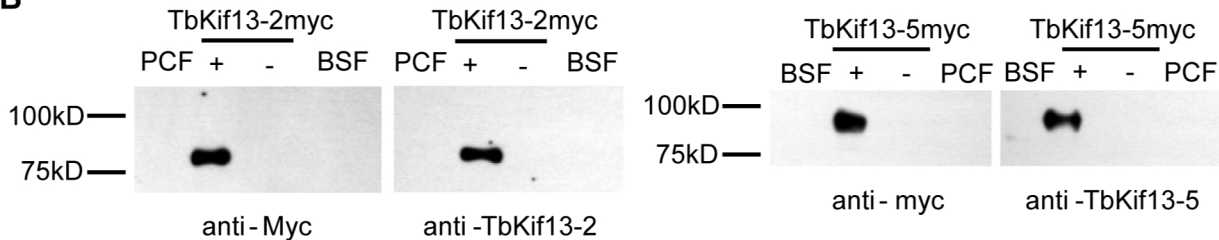

Supplement: Figure S1 — Validation of anti-kinesin antibodies. (A) Western blot using rabbit pre-immune sera (a), immune sera (b) and affinity purified antibody (c) on whole cell lysates of procyclic T. brucei. Note that the purified antibodies against TbKif13-2 and TbKif13-5 do not recognise their cognate antigen. (B) Western blot analysis using antibodies against TbKif13-2 and TbKif13-5 and anti-myc antibodies on wild-type procyclic (PCF), wild-type bloodstream (BSF) and procyclic cell lines transformed with the TbKif13-2myc or TbKif13-5myc expression vector. The lane marked (+) represents cells where expression was induced with doxycycline while the lane marked (−) represents non-induced cells. This demonstrates that the anti-TbKi13-2 and anti-TbKif13-5 antibodies do recognise their respective antigens when overexpressed from an inducible ectopic locus. (1.52 MB PDF) [file ppat.1001050.s001.pdf]

1K1N

2K1N

2K2N

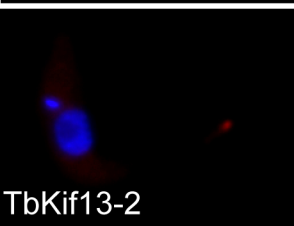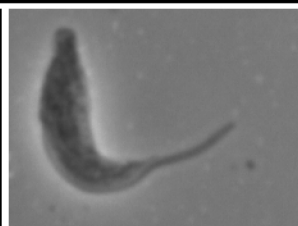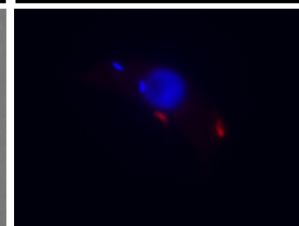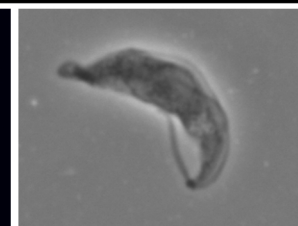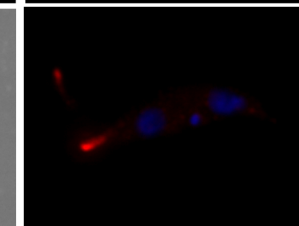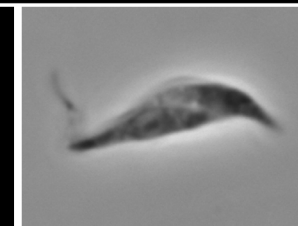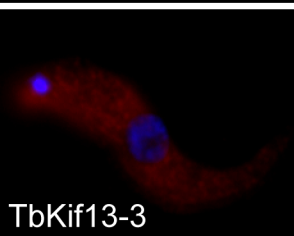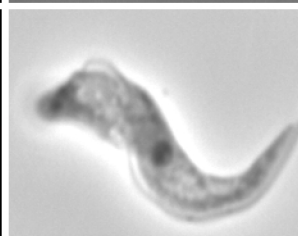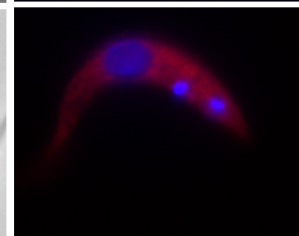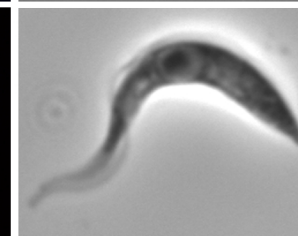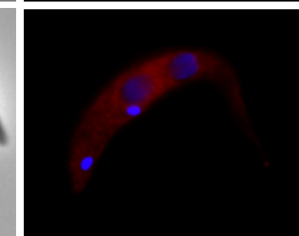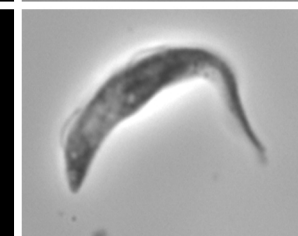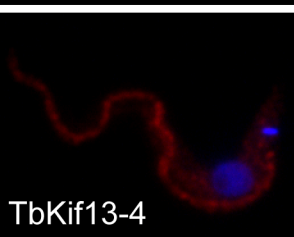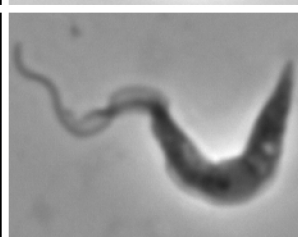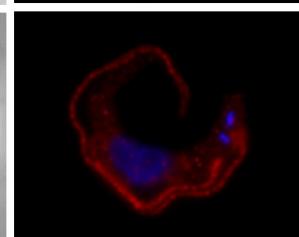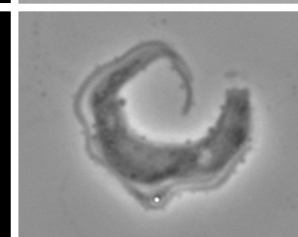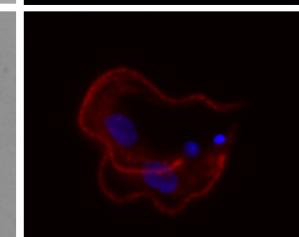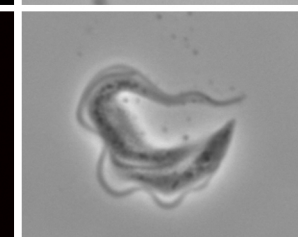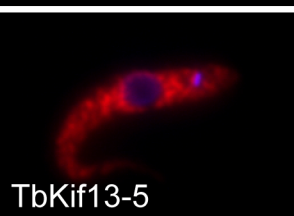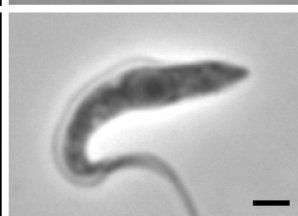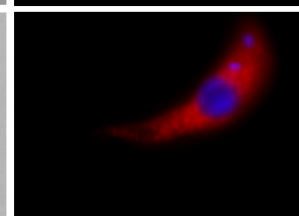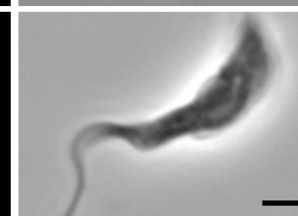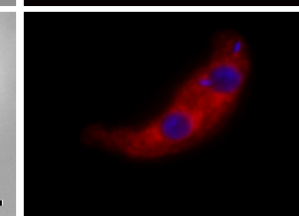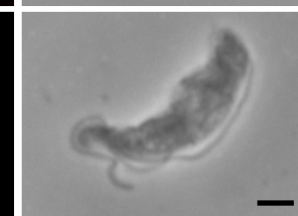

Supplement: Figure S2 — Immunolocalisation of trypanosome Kinesin-13s at different cell cycle stages (red signal). Shown are trypanosome cells in interphase (1K1N), in early (2K1N) and late mitosis (2K2N). The immunolocalisation of endogenous TbKif13-3 and TbKif13-4 was achieved using polyclonal antibodies raised against protein fragments specific to each of these two kinesins. The immunolocalisation of TbKif13-2 and TbKif13-5 was done by overexpressing a C-terminal cmyc-tagged recombinant version of the corresponding kinesins employing a tet-inducible expression system. An anti-cmyc monoclonal antibody was used for the detection of the epitope-tagged proteins. Nuclear and kinetoplast DNA has been stained with DAPI (blue). Bar, 2 µm. (3.56 MB PDF) [file ppat.1001050.s002.pdf]

TbKif13-1

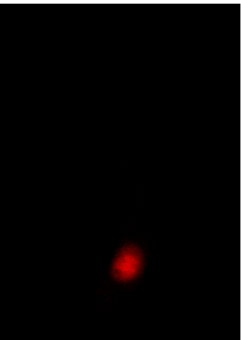

DAPI

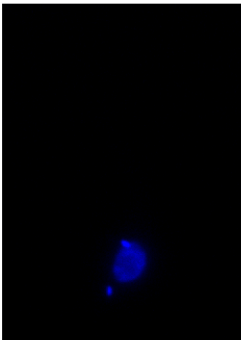

Merge

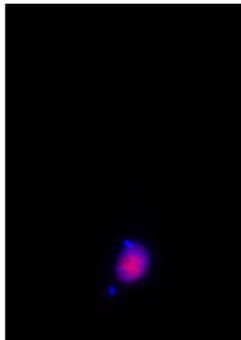

Phase

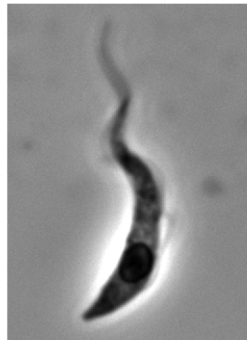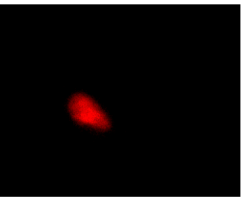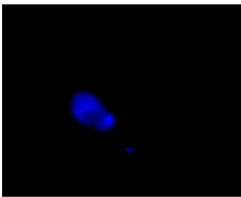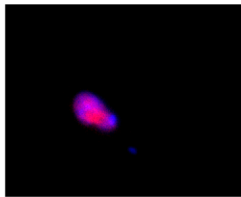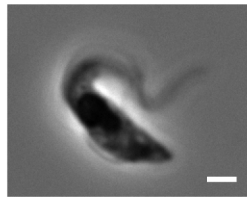

Supplement: Figure S3 — Ectopic, cmyc-tagged TbKif13-1 overexpression results in an exclusive nuclear staining. Fluorescent images of procyclic cells overexpressing a full length, ectopic copy of TbKif13-1 (red). DNA was counterstained with DAPI (blue). Bar, 2 µm. (0.98 MB PDF) [file ppat.1001050.s003.pdf]

DAPI

Telomere

Merged

-Dox

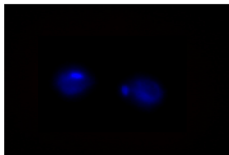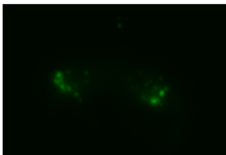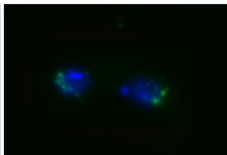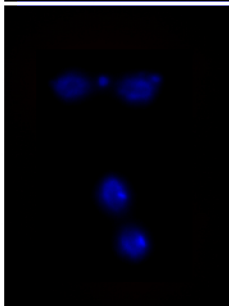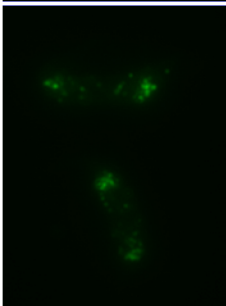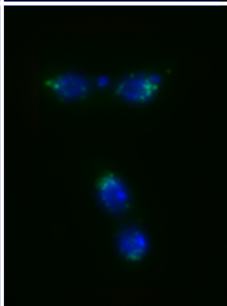

+Dox

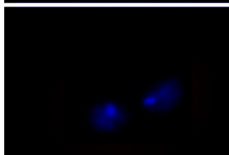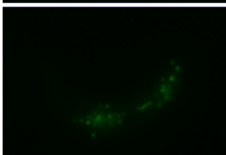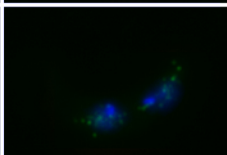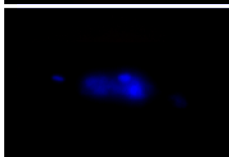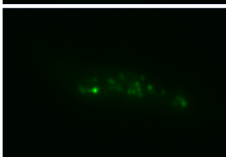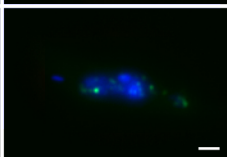

Supplement: Figure S4 — TbKif13-1 depletion results in chromosome segregation defects. Fluorescence in situ hybridisation (FISH) of procyclic cells using DNA probes (green) specific to the telomeric repeats of trypanosome chromosomes. The DNA of the kinetoplast and nucleus was stained with DAPI (blue). Shown are representative examples of induced (+Dox) and non-induced (−Dox) TbKif13-1 RNAi cells. Bar, 2 µm. (0.78 MB PDF) [file ppat.1001050.s004.pdf]

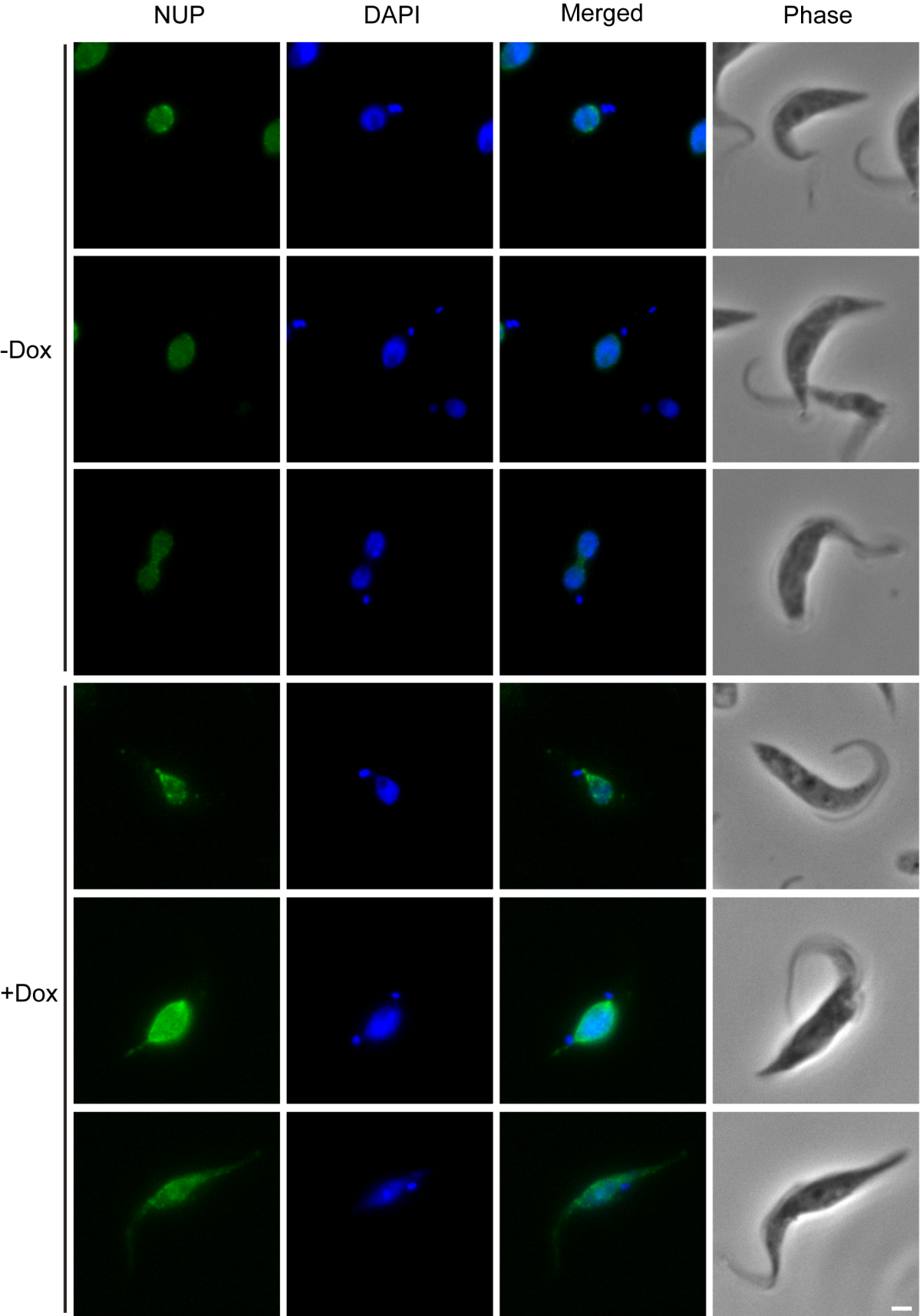

Supplement: Figure S5 — TbKif13-1 depletion results in abnormally shaped nuclei. Immunofluorescence images of induced (+Dox) and non-induced (−Dox) TbKif13-1 RNAi cells. The staining by NUP, detecting a protein of the inner nuclear envelope, is shown in green while the DNA of the kinetopast and nucleus is shown in blue. Bar, 2 µm. (3.94 MB PDF) [file ppat.1001050.s005.pdf]

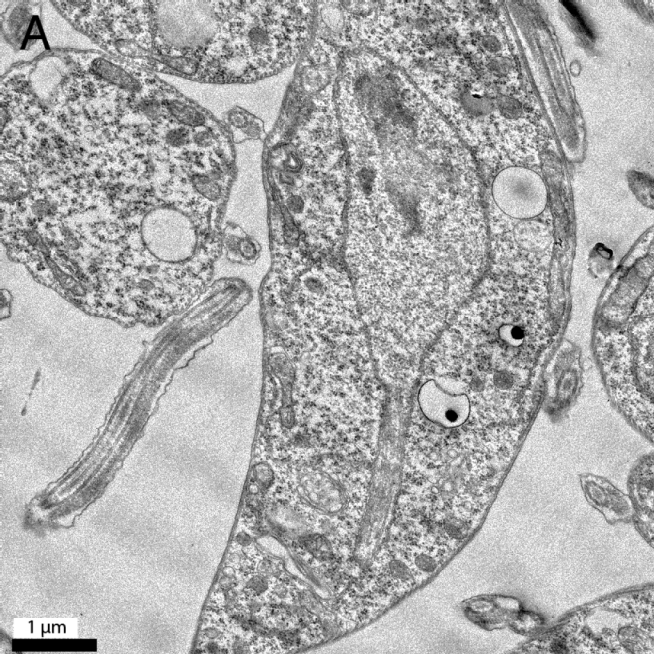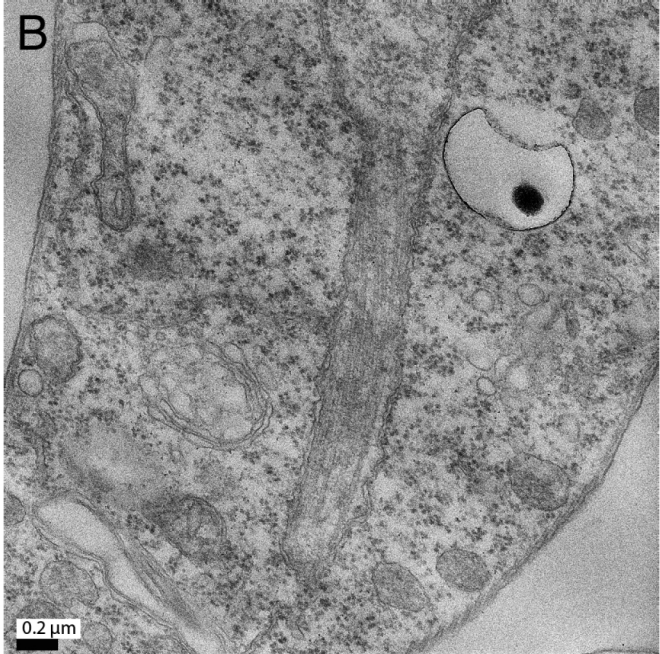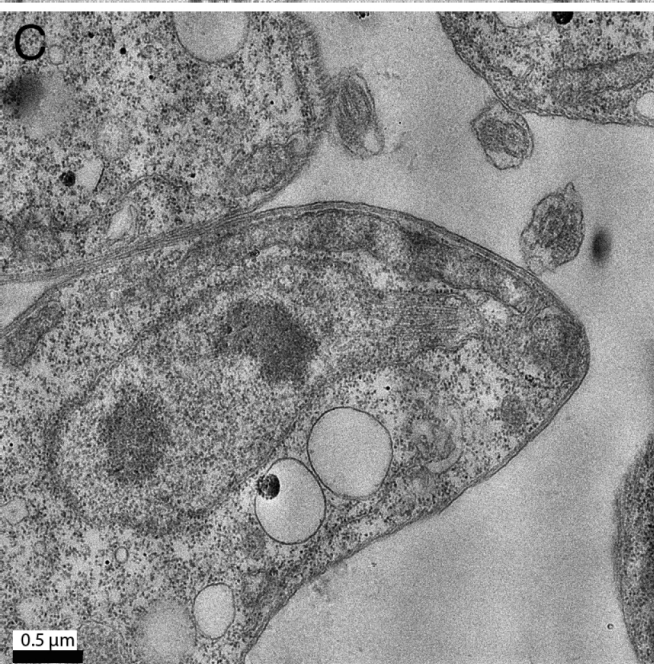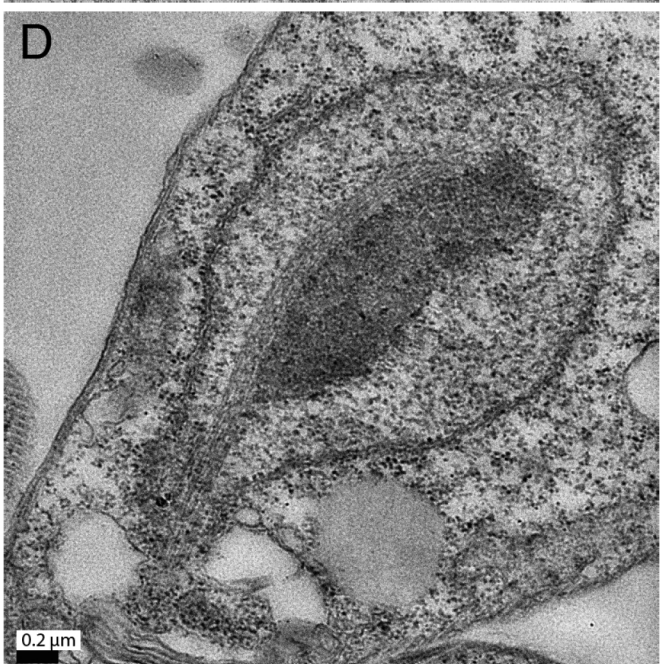

Supplement: Figure S6 — Transmission electron microscopy of TbKif13-1 RNAi depleted cells. Electron microscopy images of nuclear cross sections of TbKif13-1 depleted cells showing protrusions to its nuclear envelope (panels A–D). Panel B is an enlargement of the nuclear protrusion visible in panel A. Notice the presence of microtubule bundles in the nuclear extensions. (9.71 MB PDF) [file ppat.1001050.s006.pdf]

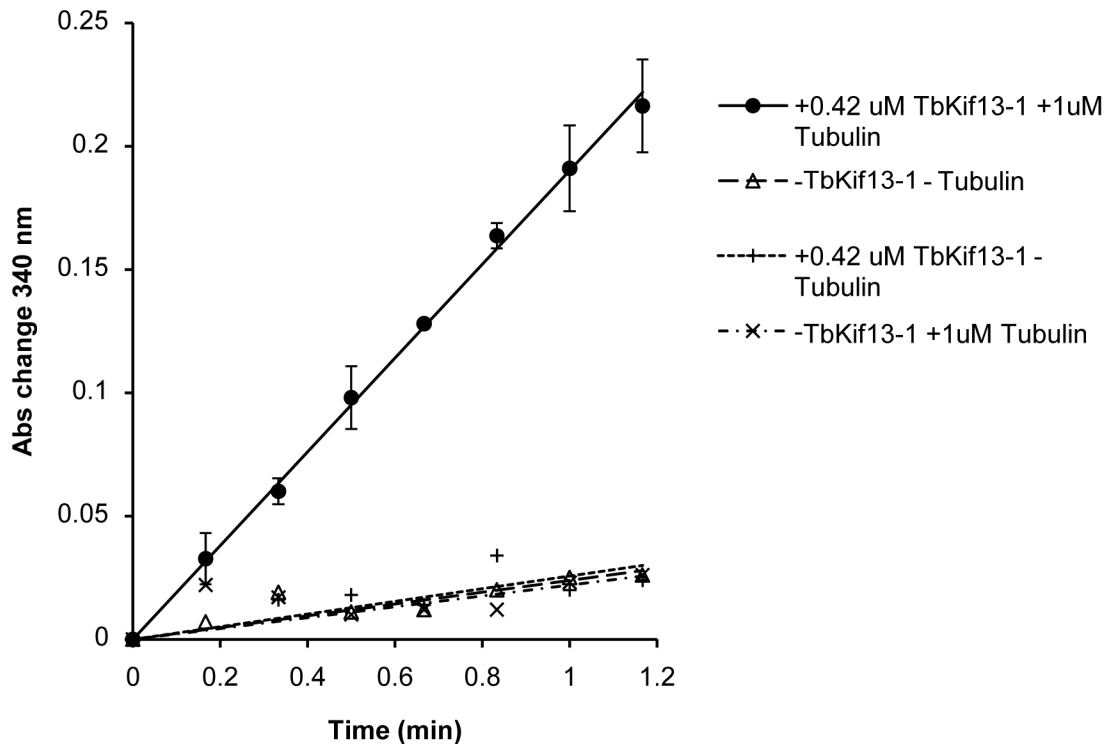

Supplement: Figure S8 — Detailed analysis of TbKif13-1 ATPase activity. The graph shows the absorbance changes of the ATPase assay over time. Rate calculations of ATPase activity for TbKif13-1 are exemplified for the presence of 1 µM tubulin. The rate of NADH depletion was obtained by measuring absorbance change at 340 nm, where one mol of oxidised NADH corresponds to the production of one mol of ADP. The ATPase rate was obtained by dividing the change of OD340 min−1 by extinction coefficient 6.22×10^−3^ µM−1 of NADH. The resulting ATPase activity was corrected by the background ATPase activity. The resulting net ATPase activity was divided by the concentration of TbKif13-1 and subsequently divided by 60 s min−1 to convert to the ATPase rate (s−1) used in Fig. 7B. Also shown in the graph are the rate of absorbance change at 340 nm of the assay in the presence and absence of TbKif13-1 and microtubules, respectively. The error bars in the graph represent the standard deviation of each data point after triplicate measurements. (0.43 MB PDF) [file ppat.1001050.s008.pdf]
