# Supplementary material for: Functional Characterisation and Drug Target Validation of a Mitotic Kinesin-13 in Trypanosoma brucei
Source: PLoS Pathog. 2010 Aug 19;6(8):e1001050. doi: 10.1371/journal.ppat.1001050 (PMC2924347; doi:10.1371/journal.ppat.1001050)
Supplement: Table S1 — Primer sequences used to generate protein expression constructs for recombinant protein production (0.16 MB PDF) [file ppat.1001050.s009.pdf]

Table S1: Primer sequences used to generate protein expression constructs for recombinant protein production.

Kinesin	Primer type	DNA sequence (5’ to 3’)
TbKif13-1	Forward	CGGGATCCCGAAACAGACATCACCAGCTGCC
	Reverse	GGAATTCCGCTCGAGACGCTGACAAAGT
TbKif13-2	Forward	CGGGATCCCGACACGATGTCCGCACTGTAG
	Reverse	GGAATTCCGCTTTGAAGCCTTAGTGGTGCC
TbKif13-3	Forward	CGGGATCCCGTCGAAACTATTCGAGGCACC
	Reverse	GGAATTCCTGGAAGAGTTCAGGGTGCAAG
TbKif13-4	Forward	CGGGATCCCGATGAGCGGTGTGCCGTCAAGAAC
	Reverse	GGAATTCCGAATTGCTTGCCCCTTACAGT
TbKif13-5	Forward	CGGGATCCCGCGGTCCTCCAGTCATCTTTC
	Reverse	GGAATTCCGGCGGTTTAGCAACTGCCTTA
