# Supplementary material for: Functional Characterisation and Drug Target Validation of a Mitotic Kinesin-13 in Trypanosoma brucei
Source: PLoS Pathog. 2010 Aug 19;6(8):e1001050. doi: 10.1371/journal.ppat.1001050 (PMC2924347; doi:10.1371/journal.ppat.1001050)
Supplement: Table S2 — Primer sequences used in the generation of cmyc-tagged kinesin expression constructs (0.16 MB PDF) [file ppat.1001050.s010.pdf]

Table S2: Primer sequences used in the generation of cmyc-tagged kinesin expression constructs.

Kinesin	Primer type	DNA sequence (5’ to 3’)
TbKif13-2	Forward	ACGGGCCCAGGGCATGACCTCACTCTGTCC
	Reverse	CGGGATCCCACGCTTTCAAGTTCATGAAGCTTTG
TbKif13-5	Forward	ACGGGCCCAGGAAATGGAGCGACAGCTTCG
	Reverse	CGGGATCCCCGGCGTTGCCGAGACTCC
