# Supplementary material for: Functional Characterisation and Drug Target Validation of a Mitotic Kinesin-13 in Trypanosoma brucei
Source: PLoS Pathog. 2010 Aug 19;6(8):e1001050. doi: 10.1371/journal.ppat.1001050 (PMC2924347; doi:10.1371/journal.ppat.1001050)
Supplement: Figure S7 — TbKif13-1 is a Kinesin-13 family member. Sequence alignment of the motor domain of TbKif13-1 against known and putative Kinesin-13 proteins. The sequences highlighted in yellow are Kinesin-13 specific motifs [1], [2]. The numbers at the left hand side of the panel denotes the relative position of the residue from the N-terminus of each corresponding protein. The corresponding GenBank accession numbers for each corresponding proteins are NP_701793 (PfKinI, Plasmodium falciparum), EAA40236 (GlKinesin-13, Giardia lamblia), BAA02165 (MmKif2, Mus musculus), AAB17358 (CgMCAK, Cricetulus griseus), NP_566534 (AtKlp3, Arabidopsis thaliana), NP_047029 (LmjKIN13-1, Leishmania major). (4.56 MB PDF) [file ppat.1001050.s007.pdf]

```
PfKinI        NLGNKIKVRSKTMNSKIKVVVRKRPLSELEKKKKDSDIITVKNNCTLYIDEPRYKVDMTKYIERHEFIVDKVFDDTVDNF 131
GlKinesin-13  VPPVDLFLN--QIQSRIRVVIRKRPINPKELSQNQRDVVTADGWNQVSIHEPKVKVDLTKYTDLHTFKFDHVFNEQSDNQ 247
MmKif2        LDYRPLTTADPIDEHRICVCVRKRPLNKKETQMKDLDVITIPSKDVVMVHEPKQKVDLTRYLENQTFRFDYAFDDSAPNE 259
CgMCAK        IECHPLTLTDPTEEHRICVCVRKRPLNKQELAKKEIDVISVPSKCLLFVHEPKLKVDLTKYLENQAFCFDFAFDETASNE 316
AtKlp3        AENLAAEKERMNAVAKIKVVVRKRPLNKKESTKNEEDIVDTHAN-CLTVHETKLKVDLTAYVEKHEFVFDAVLDEEVSND 232
LmjKIN13-1    LCRSTDTAEEVKRKSRILVAIRKRPLSAGEQTNGFTDIMDADNSGEIVLKEPKVKVDLRKYTHVHRFFFDEVFDEACDNV 198
TbKif13-1     LMRTDDSREVKRRKSRIVVAVRKRPISQCEQQRGFTDVMTTNDCDELVLAETRQKVDLTRYTHAHRFFFDEVFAETASNT 208
```

```
PfKinI        TVYENTIKPLIIDLYENGCVCSCFAYGQTGSGKTYTMLGSQP-YGQSDTPGIFQYAAGDIFTFLN-----IYDKDNTKGI 205
GlKinesin-13  EIYQYAAKPLIRSVFE-GKNCTVFAYGQTGSGKSFTMMHK--------DNGIYVLACFDILEYLRVYNGSQGNNSKFLVP 318
MmKif2        MVYRFTARPLVETIFE-RGMATCFAYGQTGSGKTHTMGGDFSGKNQDCSKGIYALAARDVFLML----KKPNYKKLELQV 334
CgMCAK        VVYRFTARPLVQTIFE-GGKATCFAYGQTGSGKTHTMGGDLSGKSQNTSKGIYAMASRDVFLLK----SQPRYRNLNLEV 391
AtKlp3        EVYRETVEPVVPLIFQ-RIKATCFAYGQTGSGKTYTM------------KPLPLKASRDILRLM----HHT-YRNQGFQL 294
LmjKIN13-1    DVYNRAARALIDTVFD-GGCATCFAYGQTGSGKTHTMLGK------GPEPGLYALAAKDMFDRL----------TSDTRI 261
TbKif13-1     DVYKRTAAALIDTVFE-GGYATCFAYGQTGSGKTHTMLGT------GGEPGIYALAAKEMLARL----------DPTKQM 271
```

```
PfKinI        FISFYEIYCGKLYDLLQKRKMVAALENGKKEVVVKDLKILRVLTKEELILKMIDGVLLRKIGVNSQNDESSRSHAILNID 285
GlKinesin-13  VVSFFEIYGGKLFDLLNNRQRLQALEDGKGNVQITGLTEKQISSVDAMLNLIDSGLTLRAVGATGANADSSRSHAILQIA 398
MmKif2        YATFFEIYSGKVFDLLNRKTKLRVLEDGKQQVQVVGLQEREVKCVEDVLKLIDIGNSCRTSGQTSANAHSSRSHAVFQII 414
CgMCAK        YVTFFEIYNGKVFDLLNKKAKLRVLEDSKQQVQVVGLQEYLVNCADDVIKMLNMGSACRTSGQTFANSNSSRSHACFQIL 471
AtKlp3        FVSFFEIYGGKLYDLLSERKKLCMREDGKQQVCIVGLQEYRVSDTDAIMELIERGSATRSTGTTGANEESSRSHAILQLA 374
LmjKIN13-1    VVSFYEIYSGKLFDLLNGRRPLRALEDDKGRVNIRGLTEHCSTSVEDLMTIIDQGSGVRSCGSTGANDTSSRSHAILEIK 341
TbKif13-1     FVSFYEIYSGKLYDLLNGRQPLRCLEDGKQNVNICGLTEHPQSNVKSIMRLIEEGTRIRSSGTTGANDTSSRSHAILEVK 351
```

```
PfKinI        LKDINKN------TSLGKIAFIDLAGSERGADTVSQNKQTQTDGANINRSLLALKECIRAMDSDK-----NHIPFRDSEL 354
GlKinesin-13  LKYTKSG------KEYSRISFIDLAGSERASDVQNSDRQTRMEGAEINKSLLALKECIRAMDKSNDSKSGAHIPFRGSKL 472
MmKif2        LRRKG--------KLHGKFSLIDLAGNERGADTSSADRQTRLEGAEINKSLLRLKECIRALGRNK-----PHTPFRASKL 481
CgMCAK        LRAKG--------RLHGKFSLVDLAGNERGADTSSADRQTRMEGAEINKSLLALKECIRALGQNK-----AHTPFRESKL 538
AtKlp3        IKKSVEGNQSKPPRLVGKLSFIDLAGSERGADTTDNDKQTRLEGAEINKSLLALKECIRALDNDQ-----GHIPFRGSKL 449
LmjKIN13-1    LKAKRTS------KQSGKFTFIDLAGSERGADTVDCARQTRLEGAEINKSLLALKECIRFLDQNR-----KHVPFRGSKL 410
TbKif13-1     LRNRGDK------ELFGKFTFIDLAGSERGADTMDCDRQTRIEGAQINKSLLALKECIRSLDLNH-----KHVPFRGSKL 420
```

```
PfKinI        TKVLRDIFVG-KSKSIMIANISPTISCCEQTLNTLRYSSRVKNFKNKSTCINEEDDTNTERISILDSKGSEMNASSIENV 433
GlKinesin-13  TMVLRDSFIG-NSQTVMIANISPNDKSCDNTLNTLRYADRVKELQHGKGGII-------------KFNVLKMGQNAADVI 538
MmKif2        TQVLRDSFIGENSRTCMIATISPGMASCENTLNTLRYANRVKEFGIS--------------------PSDIPFSQGGGSR 541
CgMCAK        TQVLRDSFIGENSRTCMIAMISPGISSCEYTLNTLRYADRVKEL--------------------------SPHSGLSGEQ 592
AtKlp3        TEVLRDSFMG-NSRTVMISCISPSSGSCEHTLNTLRYADRVKSLSKGNASKK-------------DVSSSTMNLRESTKI 515
LmjKIN13-1    TEVLRDSFIG-NCRTVMIGAVSPSNNNAEHTLNTLRYADRVKELKRNATERRTVCMPDDQEEAFFDTTESRPPSRRTTTR 489
TbKif13-1     TEVLRDSFVG-NCRTVMIGAVSPTSNSCEHTLNTLRYADRVKELKKSRSERKPLEE-NEQSEFIMEKKQTSPAARLSSHG 498
```
